# DNA fork remodeling proteins, Zranb3 and Smarcal1, are uniquely essential for aging hematopoiesis

**DOI:** 10.1111/acel.14281

**Published:** 2024-07-23

**Authors:** Saul Kushinsky, Matthew V. Puccetti, Clare M. Adams, Irina Shkundina, Nikkole James, Brittany M. Mahon, Peter Michener, Christine M. Eischen

**Affiliations:** ^1^ Department of Pharmacology, Physiology, and Cancer Biology, Sidney Kimmel Cancer Center Thomas Jefferson University Philadelphia Pennsylvania USA; ^2^ Department of Neurology Brigham and Women's Hospital, Harvard Medical School Boston Massachusetts USA

**Keywords:** aging, DNA replication stress, hematopoiesis, hematopoietic stem and progenitor cells (HSPC), Smarcal1, Zranb3

## Abstract

Over a lifetime, hematopoietic stem and progenitor cells (HSPCs) are forced to repeatedly proliferate to maintain hematopoiesis, increasing their susceptibility to DNA damaging replication stress. However, the proteins that mitigate this stress, protect HSPC replication, and prevent aging‐driven dysregulation are unknown. We report two evolutionarily conserved, ubiquitously expressed chromatin remodeling enzymes with similar DNA replication fork reversal biochemical functions, Zranb3 and Smarcal1, have surprisingly specialized roles in distinct HSPC populations. While both proteins actively mitigate replication stress and prevent DNA damage and breaks during lifelong hematopoiesis, the loss of either resulted in distinct biochemical and biological consequences. Notably, defective long‐term HSC function, revealed with bone marrow transplantation, caused hematopoiesis abnormalities in young mice lacking Zranb3. Aging significantly worsened these hematopoiesis defects in *Zranb3*‐deficient mice, including accelerating the onset of myeloid‐biased hematopoietic dysregulation to early in life. Such *Zranb3*‐deficient HSPC abnormalities with age were driven by accumulated DNA damage and replication stress. Conversely, Smarcal1 loss primarily negatively affected progenitor cell functions that were exacerbated with aging, resulting in a lymphoid bias. Simultaneous loss of both Zranb3 and Smarcal1 compounded HSPC defects. Additionally, HSPC DNA replication fork dynamics had unanticipated HSPC type and age plasticity that depended on the stress and Zranb3 and/or Smarcal1. Our data reveal both Zranb3 and Smarcal1 have essential HSPC cell intrinsic functions in lifelong hematopoiesis that protect HSPCs from replication stress and DNA damage in unexpected, unique ways.

## Graphical abstract

Description: DNA fork reversal proteins, Zranb3 and Smarcal1, have hematopoietic stem and progenitor cell (HSPC)‐specific functions that mitigate DNA replication stress and DNA damage to protect lifelong hematopoiesis. Zranb3 loss causes an accelerated aging myeloid‐biased phenotype due to long‐term HSC and progenitor cell defects. Smarcal1 loss causes progenitor cell disfunction and an atypical lymphoid bias.

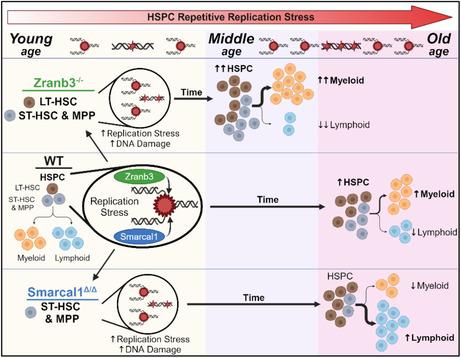

## 1 INTRODUCTION

During hematopoiesis, hematopoietic stem and progenitor cells (HSPCs) proliferate and differentiate into all mature blood cells (Olson et al., 2020). HSPCs are comprised of multipotent and lineage‐committed oligopotent cell types that respond to hematopoietic system insults (Pietras et al., 2015). The earliest hematopoietic progenitors include multipotent progenitor populations (MPPs), lineage‐biased cells capable of quickly differentiating, and short‐term hematopoietic stem cells (ST‐HSCs), which differentiate into MPPs. HSPCs and hematopoiesis, especially lifelong hematopoiesis, are maintained by long‐term HSCs (LT‐HSCs), a rare population that is the only hematopoietic cell type with true self‐renewal capabilities (Olson et al., 2020; Wilson et al., 2008). However, as LT‐HSCs age they functionally exhaust, becoming quiescent and unable to reactivate (Beerman et al., 2014; Rossi et al., 2007). Remaining LT‐HSCs show signs of dysfunction, including reduced progeny output and myeloid‐bias, resulting in increased myeloid and decreased lymphoid populations (Purton, 2022; Rossi et al., 2008). The changes that drive hematopoietic aging, and the mechanisms that protect against those alterations are complex and poorly understood.

Environmental exposures (e.g., infections, irradiation, chemotherapy) and bleeding lead to hematopoietic system insults requiring proliferative bursts of HSPCs (Flach & Milyavsky, 2018; King & Goodell, 2011). Proliferation, however, leads to DNA replication stress, that when unresolved can result in DNA damage and genomic instability (Berti et al., 2020; Walter et al., 2015). Mutations in HSCs can be particularly dangerous, as they are passed to all downstream populations, potentially impacting their function and survival (Olson et al., 2020). To limit DNA damage and maintain healthy HSCs, LT‐HSCs remain predominantly dormant (Li et al., 2022; Walter et al., 2015). Despite this, aging LT‐HSCs accumulate marks of DNA damage (Beerman et al., 2014; Flach et al., 2014; Rossi et al., 2007; Rube et al., 2011). It is currently unclear how age‐related changes affect the DNA replication stress responses of LT‐HSCs and downstream progenitor populations including ST‐HSCs and MPPs. Thus, it is critical to understand which DNA replication stress response mechanisms specifically protect HSPCs and hematopoiesis.

During DNA replication stress, many reported that stalled DNA replication forks underwent fork reversal to stabilize the DNA while the problem is mitigated (Neelsen & Lopes, 2015; Quinet et al., 2017). Recently, we and others showed that responses can instead activate replication fork repriming, an alternative mechanism that bypasses DNA lesions that stall replication forks, to quickly continue DNA synthesis, but this can lead to DNA damage (Bianchi et al., 2013; Garcia‐Gomez et al., 2013; Jacobs et al., 2022; Mouron et al., 2013; Quinet et al., 2020). There is also indirect evidence that in mice and humans stalled forks can be bypassed by new origin firing (Saxena & Zou, 2022). However, the conditions under which a specific mechanism is chosen are unclear. Using mouse models, we reported that Zranb3 and Smarcal1, evolutionarily conserved, closely‐related fork reversal proteins that protect replication forks and mitigate DNA replication stress (Betous et al., 2012; Ciccia et al., 2012; Poole & Cortez, 2017), are essential to the oncogene‐induced DNA replication stress response in pre‐B cells (Puccetti et al., 2019). In addition, we showed Smarcal1 is critical for stressed hematopoiesis induced by γ‐irradiation and chemotherapy (Puccetti et al., 2017). However, neither Smarcal1 nor Zranb3 appear to be essential for the acute HSPC response to polyinosinic:polycytidylic acid (pI:pC) insult that mimics a viral infection. Instead, after pI:pC, HSPCs favored replication fork repriming (Jacobs et al., 2022). These data suggest much remains unknown about the DNA replication stress response in hematopoietic cells, as it may be dependent on the cell type, the insult causing the replication stress, and/or other factors.

In this study, our investigations into the mechanisms that protect HSPC DNA replication during normal, stressed, and aging hematopoiesis reveal previously unknown contributions of Zranb3 and Smarcal1 in mitigating physiological DNA replication stress and preventing its consequences. Unexpectedly, HSPCs, especially after aging, had population‐specific stress responses, utilizing Zranb3 and/or Smarcal1 in distinct capacities. Furthermore, LT‐HSC function was protected by Zranb3, but not Smarcal1, leading to an accelerated aging myeloid‐biased phenotype with Zranb3 loss. Our data elucidate that stressed and lifelong hematopoiesis rely on the fundamentally unique, nonredundant activities of Zranb3 and Smarcal1 and uncover new insights into biologically relevant DNA replication stress response mechanisms.

## 2 RESULTS

### 2.1 Loss of Zranb3 or Smarcal1 confers a competitive disadvantage to HSPCs

**FIGURE 1:**
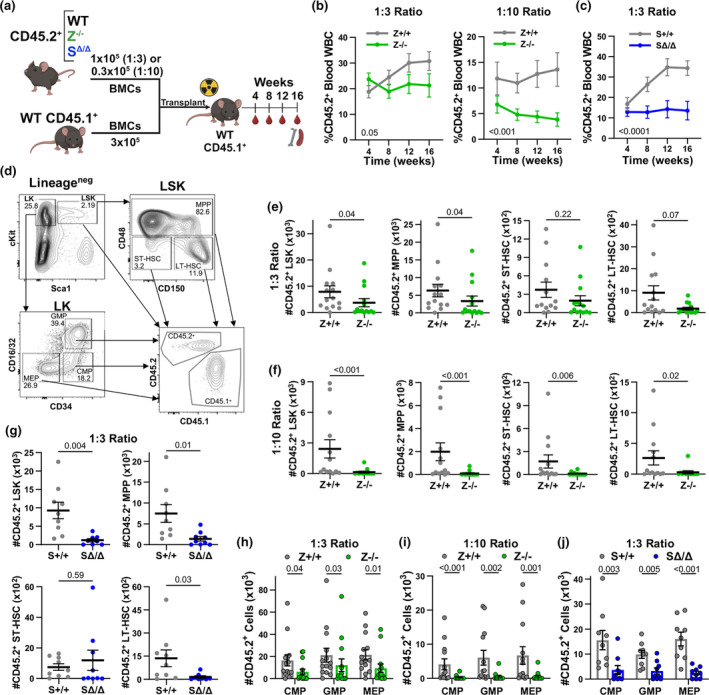
Both Zranb3 and Smarcal1 protect HSPC function. (a) Schematic of competitive BMTs to test HSPC function. (b, c) Donor chimerism in peripheral blood at 4 week intervals after competitive BMT of CD45.2^+^ total bone marrow donor cells (BMCs) from *Zranb3* ^*−/−*^ (b) or *Smarcal1* ^*Δ/Δ*^ (c) and their respective wild‐type (WT) littermates mixed with WT CD45.1^+^ competitor BMCs in a 1(CD45.2):3(CD45.1) ratio (b left, c) or 1(CD45.2):10(CD45.1) ratio (b right) injected into lethally‐irradiated WT CD45.1^+^ recipients. (mean ± SEM; p‐value determined by two‐way ANOVA). (d) Representative HSPC gating strategy used for BMT recipients. (e–j) Numbers of CD45.2^+^ HSPCs/femur in the bone marrow of the donor genotypes indicated after 16 weeks (mean ± SEM; symbols denote individual recipients; p‐values determined as described in the methods). For all, at least 8 recipients/genotype evaluated.

HSPCs undergo tremendous proliferation pressure to maintain hematopoiesis, but how their DNA replication stress is mitigated is unclear. Zranb3 and Smarcal1 are important components of the stress response in highly proliferating cells, (Poole & Cortez, 2017), but their role in HSPCs, despite ubiquitous expression (Heng et al., 2008), remain unknown. To investigate the contribution of Zranb3 and Smarcal1 to hematopoiesis, we performed bone marrow transplantation (BMT), the gold standard for evaluating biological HSPC function and proliferative ability. Competitive BMTs with bone marrow cells (BMCs) lacking Zranb3 or Smarcal1 were conducted by mixing CD45.2^+^ *Zranb3* ^*−/−*^, *Smarcal1* ^Δ/Δ^, or littermate‐matched wild‐type BMCs with CD45.1^+^ wild‐type BMCs at a 1:3 ratio, or the more challenging 1:10 ratio, and transplanted into lethally‐irradiated CD45.1^+^ recipients (Figure 1a). Over 16 weeks, mice that received *Zranb3* ^*−/−*^ or *Smarcal1* ^*Δ/Δ*^ BMCs had reduced CD45.2^+^ blood leukocytes (Figure 1b,c; Figure S1A,B). After 16 weeks, recipients of *Zranb3* ^−/−^ BMCs (1:10 ratio) and *Smarcal1* ^Δ/Δ^ BMCs (1:3 ratio) showed reduced splenic CD45.2^+^ WBCs (B‐cells, T‐cells, and neutrophils; Figure S1A,B). Bone marrow also had reduced donor BMCs in all transplants (Figure S1A,B). To determine if the differences in CD45.2^+^ mature populations were due to defects in progenitors (Pietras et al., 2015), we evaluated the HSPC compartment (Figure 1d; Figure S1C). We observed significantly reduced lineage‐negative Sca1^+^cKit^+^ cells (LSK), MPPs, granulocyte‐monocyte progenitors (GMP), common myeloid progenitors (CMP), and megakaryocyte‐erythroid progenitors (MEP) in all transplants (Figure 1e–j). In recipients of *Zranb3* ^−/−^ BMCs, there were also reductions in CD45.2^+^ LT‐HSCs and ST‐HSCs (Figure 1e,f), whereas recipients of *Smarcal1* ^*Δ/Δ*^ BMCs had decreased CD45.2^+^ LT‐HSCs but not ST‐HSCs (Figure 1g). Together, these data indicate that both *Zranb3*‐ and *Smarcal1*‐deficient HSPCs have a competitive disadvantage, leading to multi‐lineage global defects of hematopoietic populations after BMT.

To ensure our results were not due to an altered ability of *Zranb3* ^−/−^ or *Smarcal1* ^*Δ/Δ*^ HSCs to traffic to the bone marrow upon BMT, we evaluated HSC homing. BMCs from *Zranb3*‐ or *Smarcal1*‐deficient mice and wild‐type littermate‐matched donors were labeled with carboxyfluorescein succinimidyl ester (CFSE) and transplanted. There was no difference in transplanted HSCs present in the bone marrow for either genotype (Figure S1D). Therefore, the BMT data indicate loss of Zranb3 or Smarcal1 does not impact HSC ability to reach the bone marrow, but once there, it has a competitive disadvantage against wild‐type HSPCs.

### 2.2 Zranb3 and Smarcal1 protect young HSPCs from DNA replication stress and DNA damage

**FIGURE 2:**
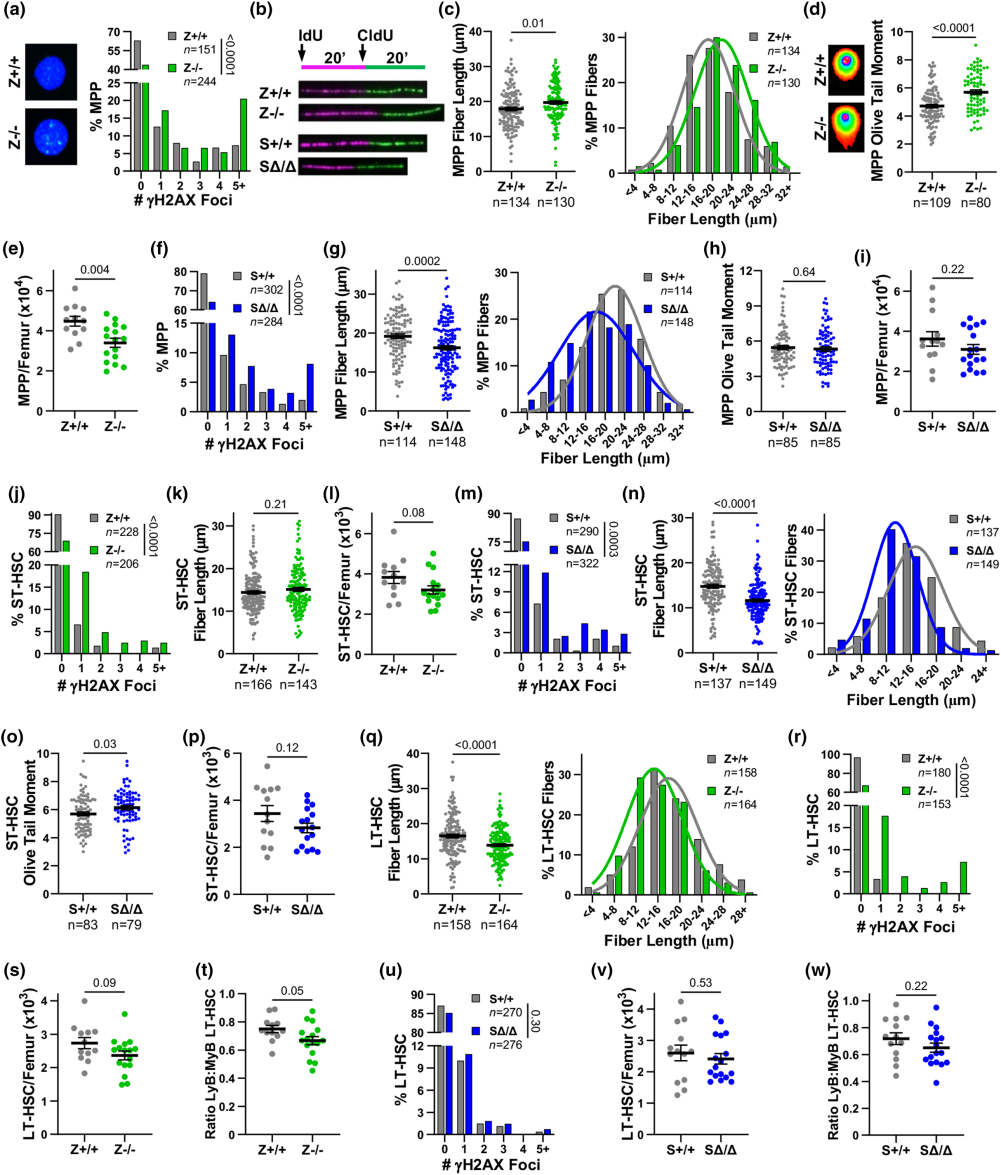
HSPC defects arise in young mice without Zranb3 or Smarcal1 due to excess DNA replication stress. (a–i) MPPs were isolated (a–d, f–h) or measured (e, i) by flow cytometry from littermates (genotype indicated). Percentage of cells with the indicated γH2AX foci/cell of isolated MPPs (a,f) by immunofluorescence. Representative images in (a). DNA fiber labelling assay design and representative images of fiber tracks from isolated MPPs (b). DNA fiber labelling in isolated MPPs (c, g). DNA fiber lengths (left) and binning of fiber lengths (right). Comet assay Olive tail moments from MPPs (d, h). Representative images in (d). Number of MPPs/femur (e, i) as measured by flow cytometry. (j–p) ST‐HSCs were isolated (j, k, m–o) or measured (l, p) by flow cytometry from littermates (genotypes indicated). Percentage of cells with the indicated γH2AX foci/cell in ST‐HSCs (j, m) by immunofluorescence. DNA fiber labelling in isolated ST‐HSCs (k, n). DNA fiber lengths (k, n left) and binning of fiber lengths (n right). Number of ST‐HSCs/femur (l, p) as measured by flow cytometry. Comet assay Olive tail moments from ST‐HSCs (o). (q–w) LT‐HSCs from littermates (genotypes indicated) were isolated (q, r, u) or measured (s, t, v, w) by flow cytometry (see Figure S2A). LT‐HSC DNA fiber labelling (q). DNA fiber lengths (left) and binning of fiber lengths (right). Percentage of LT‐HSCs with the indicated γH2AX foci/cell (r, u; immunofluorescence). Number of LT‐HSCs/femur (s, v) and the ratio of LyB‐LT‐HSCs to MyB‐LT‐HSCs (t, w). For (a, f, j, m, r, u) “*n*” denotes numbers of cells; *p*‐value determined by chi‐squared test for trend. For (c, g, k, n, q), “*n*” denotes number of fibers; symbols denote individual fibers; mean ± SEM; *p*‐values determined by Student's *t*‐tests; curves are calculated Gaussian distribution. For (d, h, o) “*n*” is number of cells; symbols denote individual cells; mean ± SEM; *p*‐values determined by Student's *t*‐tests. Representative experiments shown for (a, c, d, f–h, j, k‐o, q, r, and u). For (e, i, l, p, s, t, v, and w), 12–17 mice/genotype; symbols denote individual mice; mean ± SEM; *p*‐values determined by Student's *t*‐test.

Defects after BMT were observed early during reconstitution with BMCs lacking Zranb3 or Smarcal1, suggesting impaired DNA replication stress and damage responses in the multi‐lineage capable LSK hematopoietic progenitor populations. Therefore, we evaluated MPPs (Figure S2A; (Pietras et al., 2015)), which maintain immediate hematopoiesis (Benveniste et al., 2010; Olson et al., 2020; Purton, 2022), in unstressed, non‐transplanted young (6‐8‐week‐old) mice. In *Zranb3* ^−/−^ MPPs, we observed increased DNA damage (γH2AX foci) compared to littermates (Figure 2a). To determine the source of the DNA damage, we evaluated DNA replication fork dynamics using the DNA fiber labeling assay (Figure 2b). We detected increased fork lengths (Figure 2c), indicating increased replication stress that activated an alternative bypass response, not fork stalling. As unresolved DNA replication stress can result in DNA breaks, we performed comet assays, which showed *Zranb3* ^−/−^ MPPs had increased DNA breaks (Figure 2d). Consequently, LSKs and MPPs in *Zranb3* ^*−/−*^ mice were significantly reduced compared to littermate controls (Figure 2e; Figure S2B). Similar to Zranb3 loss, *Smarcal1*‐deficient MPPs showed increased DNA damage (γH2AX foci, Figure 2f) and DNA replication stress (Figure 2g). However, there were decreased DNA fiber track lengths in *Smarcal1* ^Δ/Δ^ MPPs, indicating DNA fork stalling, but this did not lead to more DNA breaks (Figure 2h), and there was only a slight trend toward reduced MPPs in mice lacking Smarcal1 (Figure 2i; Figure S2C; summarized in Table S1). Therefore, in MPPs lacking Zranb3, replication stress activates a bypass mechanism that is prone to DNA damage/breaks, resulting in reduced MPP populations during normal hematopoiesis and following transplantation (summarized in Table S1). Conversely, normal hematopoiesis without Smarcal1 induces MPP DNA replication fork stalling, which should facilitate DNA repair and production of MPPs, suggesting Smarcal1 is necessary but appears not to be required under unstressed conditions. In contrast, Smarcal1 is required during transplantation‐induced stressed hematopoiesis, as either *Smarcal1*‐deficient MPPs or an earlier progenitor had diminished capabilities to respond to DNA replication stress and produce progeny.

To determine if the effects of Zranb3 or Smarcal1 loss impacted earlier progenitors, we evaluated ST‐HSCs, which maintain intermediate hematopoiesis (Benveniste et al., 2010; Purton, 2022), in unstressed young mice. Again, we observed more DNA damage (γH2AX foci) with Zranb3 loss (Figure 2j). While there were no differences in DNA fiber lengths or breaks (Figure 2k; Figure S2D,E), there was a trend toward decreased ST‐HSC numbers (Figure 2l; Table S1). In contrast, ST‐HSCs lacking Smarcal1 had both increased DNA damage (γH2AX foci; Figure 2m) and DNA fork stalling (decreased fiber lengths; Figure 2n), suggesting a greater importance for Smarcal1 in this population. Consistent with a small increase in DNA breaks (Figure 2o), Smarcal1 loss did not significantly reduce (a trend) ST‐HSC numbers (Figure 2p; Table S1). Therefore, ST‐HSCs lacking Smarcal1 had more DNA replication stress than *Zranb3* ^*−/−*^ ST‐HSCs, which could affect downstream MPPs, particularly under replication stress conditions, such as transplantation.

### 2.3 Significant LT‐HSC functional defects arise in cells deficient in Zranb3, but not Smarcal1

Because long‐term hematopoiesis is maintained by self‐renewing LT‐HSCs, we next investigated the contribution of Zranb3 and Smarcal1 to LT‐HSCs. Zranb3 loss in LT‐HSCs from young unstressed mice caused reduced DNA fiber lengths (Figure 2q), indicating increased DNA replication stress, and elevated DNA damage (Figure 2r; reviewed in Table S1). There was both a trend toward increased DNA breaks (Figure S2F) and decreased LT‐HSC numbers (Figure 2s). Previous studies characterized LT‐HSCs into healthy, balanced “Lymphoid‐Biased” (LyB)‐LT‐HSCs and unbalanced “Myeloid‐Biased” (MyB)‐LT‐HSCs based on cell surface markers and progeny produced (Beerman et al., 2010; Morita et al., 2010). Interestingly, further classification of *Zranb3*‐deficient LT‐HSCs revealed reduced healthy LyB‐LT‐HSCs and increased MyB‐LT‐HSCs (Figure 2t; Figure S2G; Table S1). In stark contrast, Smarcal1 loss in LT‐HSCs resulted in no difference in DNA damage (Figure 2u) or DNA breaks (Figure S2H), and there were similar LT‐HSC numbers (Figure 2v) and normal ratios of LyB‐LT‐HSCs to MyB‐LT‐HSCs (Figure 2w; Table S1). Thus, in young, unstressed mice, Zranb3 is essential to mitigate LT‐HSC DNA replication stress, protect from DNA damage, and maintain healthy LT‐HSCs, but Smarcal1 appears dispensable.

To ensure that the differences observed with loss of Zranb3 or Smarcal1 are due to cell intrinsic effects and not the bone marrow niche, which is critical for hematopoiesis, we tested the ability of the niche to maintain hematopoiesis in the absence of either protein. We transplanted CD45.1^+^ wild‐type BMCs into lethally‐irradiated littermate‐matched CD45.2^+^ wild‐type, *Zranb3* ^−/−^, or *Smarcal1* ^Δ/Δ^ mice (Figure S3A). Blood, spleen, and bone marrow populations, including HSPCs, showed no differences in reconstitution by CD45.1^+^ wild‐type cells (Figure S3B–G). Therefore, neither Zranb3 nor Smarcal1 affects the ability of the bone marrow niche to maintain hematopoiesis, but instead has HSC or downstream progenitor cell intrinsic effects.

**FIGURE 3:**
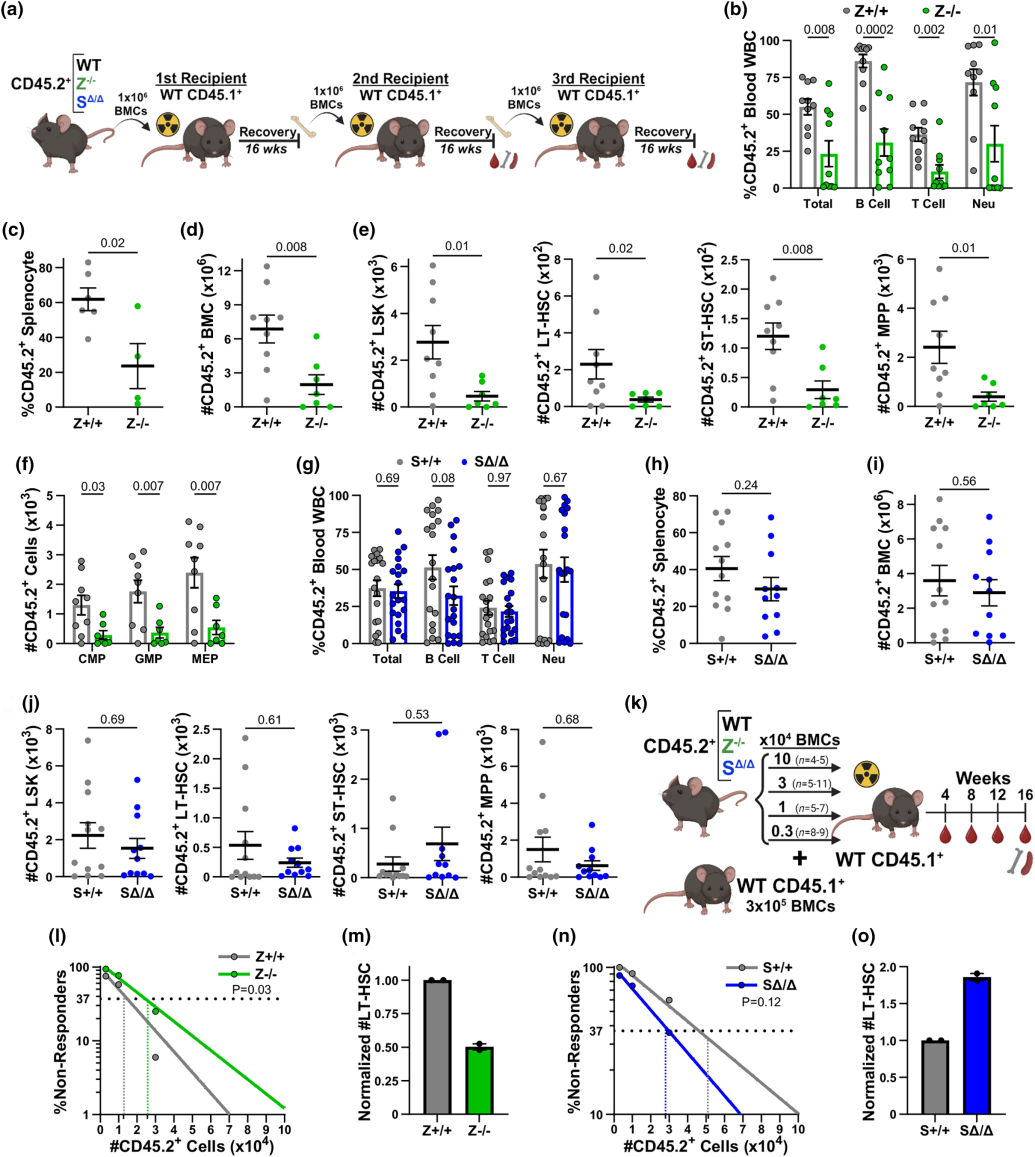
Loss of Zranb3, but not Smarcal1, negatively impacts LT‐HSC function. (a) Schematic of serial BMTs to evaluate LT‐HSC function. (b–j) After 16 weeks post‐tertiary transplant, donor chimerism in peripheral blood (b, g; ≥10 recipients/genotype), splenocytes (c, h; ≥4 recipients/genotype), and donor total BMCs (d, i) or HSPCs/femur (e, f, j; ≥7 recipients/genotype) of the indicated donor genotypes (mean ± SEM; symbols denote individual recipients; *p*‐values determined as described in the methods). (k) Schematic of limiting dilution transplantation assay to determine the frequency of functional LT‐HSCs. Decreasing numbers of CD45.2^+^ total BMCs were mixed with 3 × 10^5^ CD45.1^+^ total BMCs and transplanted into lethally‐irradiated CD45.1^+^ recipients, “*n*” denotes number of recipients/transplant. (l–o) Quantification of the percentage of recipient mice containing <1% donor‐derived cells (% non‐responders) in the peripheral blood 16 weeks posttransplant of the indicated cell dose and donor genotypes (l, n; HSC frequencies and p‐values of 2 pooled independent experiments determined by 2‐tailed ratio of proportions calculated by L‐Calc). Ratio of the frequency of functional *Zranb3* ^*−/−*^ (m) and *Smarcal1* ^*Δ/Δ*^ (o) LT‐HSCs normalized to the frequency of wild‐type LT‐HSCs (mean ± SEM of two independent experiments).

To further test the requirements of Zranb3 in LT‐HSC function, we performed three rounds of serial BMTs (Purton & Scadden, 2007) with 16 weeks of recovery between each transplant (Figure 3a). Post tertiary serial BMT of *Zranb3* ^−/−^ BMCs, mice demonstrated multi‐lineage deficits of CD45.2^+^ populations in the blood, spleen, and bone marrow (Figure 3b–d; Figure S3H). In the HSPC compartment, there were significantly reduced multi‐lineage LSKs (LT‐HSCs, ST‐HSCs, MPPs, Figure 3e) and lineage‐committed progenitors (CMPs, GMPs, MEPs, Figure 3f). In contrast, after tertiary transplant of *Smarcal1* ^*Δ/Δ*^ BMCs, CD45.2^+^ populations in the blood, spleen, and bone marrow, including the HSPC and LSK compartments, were not statistically significantly different compared to controls (Figure 3g–j; Figure S3I). These results strongly suggest Zranb3, but not Smarcal1, is essential for LT‐HSC function to repetitively reconstitute the bone marrow.

For additional, rigorous evaluation of LT‐HSC function, we performed limiting dilution BMTs (Purton & Scadden, 2007) to measure the frequency of functional LT‐HSCs (Figure 3k). *Zranb3* ^−/−^ BMCs had decreased frequencies (Figure 3l, m), whereas *Smarcal1* ^Δ/Δ^ BMCs had increased frequencies (Figure 3n, o) of functional HSCs. Therefore, Zranb3 loss impacts functional HSCs, which leads to a reduced ability to maintain long‐term hematopoiesis. In stark juxtaposition, hematopoiesis without Smarcal1 appears to result in a compensatory increase in functional HSCs to counteract defects in downstream progenitor cells.

### 2.4 LT‐HSC functional exhaustion with Zranb3 loss results in myeloid bias, an aging phenotype

**FIGURE 4:**
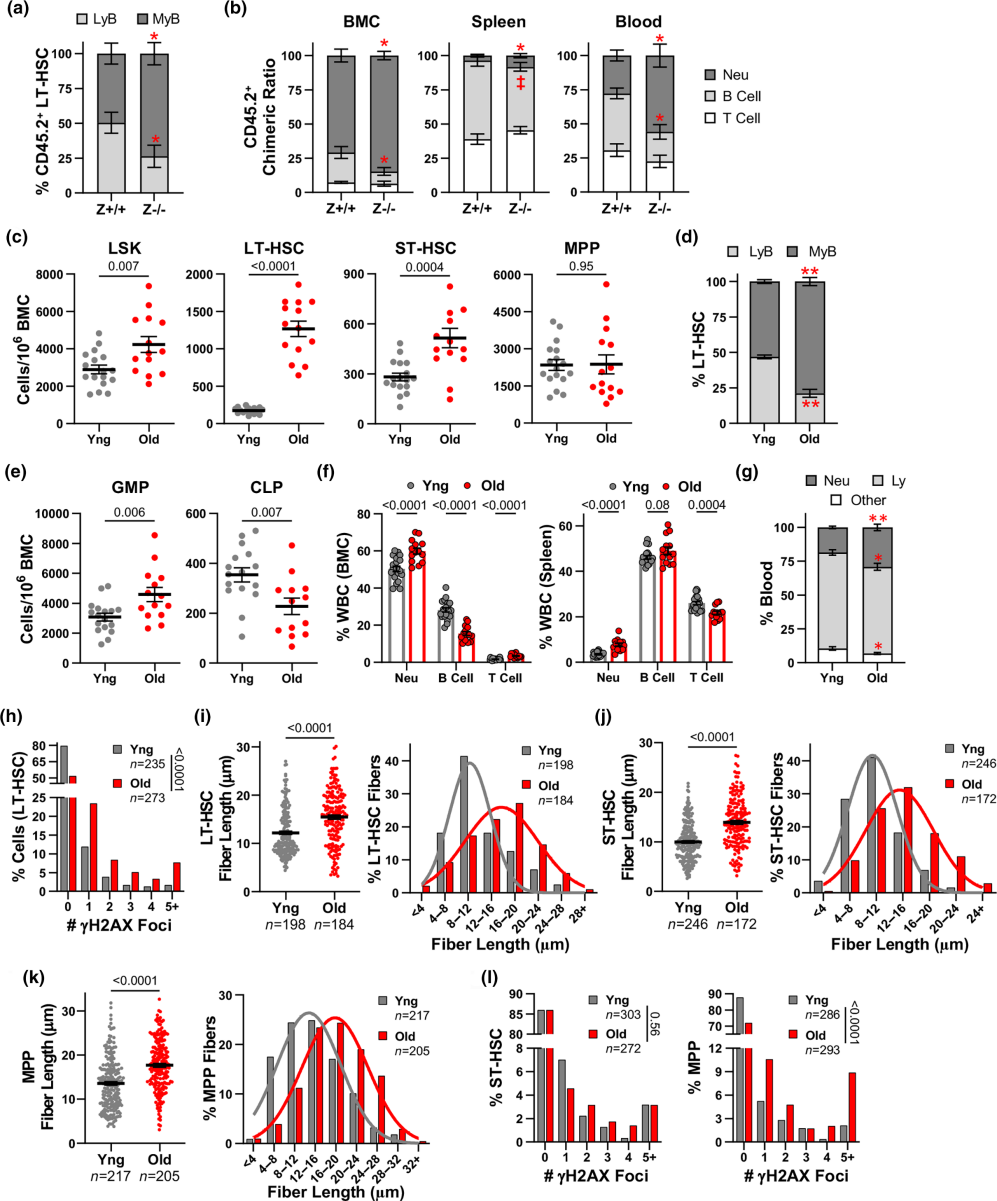
Hematopoietic alterations and HSPC dysfunction with LT‐HSC functional exhaustion. (a, b) Secondary transplant recipients (see Figure 3a) of *Zranb3* ^*−/−*^ BMCs were evaluated by flow cytometry and percentages of LyB‐LT‐HSCs and MyB‐LT‐HSCs out of total CD45.2^+^ LT‐HSCs (a) or chimeric ratio of specific CD45.2^+^ populations in the bone marrow (b, left), spleen (b, center), and blood (b, right) were determined (5–7 recipients/genotype). (c–g) Hematopoietic populations determined by flow cytometry from young (Yng) and old wild‐type mice. The frequency of the indicated HSPC type/10^6^ BMCs (c, e). Percentages of LyB‐LT‐HSCs and MyB‐LT‐HSCs of total LT‐HSCs in young and old mice (d). Percentage of the specific populations of white blood cells (WBCs) in bone marrow (f, left), spleen (f, right), and blood (g). At least 11 mice evaluated/age; symbols denote individual mice. For (a–g), mean ± SEM; Neu, neutrophil; Ly, lymphocyte; *p*‐values determined by Student's *t*‐test; ‡*p* = 0.06, *p ≤ 0.05, **p < 0.0001. (h–l) LT‐HSCs (h, i), ST‐HSCs (j, l left), and MPPs (k, l right) were isolated by flow cytometry from young and old wild‐type mice. Percentage of cells with the indicated γH2AX foci/cell (immunofluorescence) of LT‐HSCs (h), ST‐HSCs (l, left), or MPPs (l, right); *p*‐values determined by chi‐squared test for trend. DNA fiber lengths (i–k left; mean ± SEM) and binning of fiber lengths (i–k right) in isolated LT‐HSCs (i), ST‐HSCs (j), and MPPs (k); “*n*” denotes number of fibers; symbols denote individual fibers; *p*‐values determined by Student's *t*‐test; curves are calculated Gaussian distribution.

Since Zranb3 loss reduced hematopoietic reconstitution after serial BMT, an indicator of accelerated LT‐HSC functional exhaustion, we evaluated secondary BMT recipients (Figure 3a) to determine whether progeny of partially exhausted LT‐HSCs are dysregulated. Mice that received *Zranb3* ^*−/−*^ BMCs had significantly decreased LyB‐LT‐HSCs and increased MyB‐LT‐HSCs (Figure 4a), leading to increased neutrophil and decreased lymphocytes in the bone marrow, spleen, and blood (Figure 4b). Conversely, recipients of *Smarcal1* ^*Δ/Δ*^ BMCs showed no significant differences in LyB‐ and MyB‐LT‐HSCs or biases in mature populations (Figure S4A,B). Thus, after serial transplantation in the absence of Zranb3, but not Smarcal1, LT‐HSCs are impaired, produce myeloid‐biased progeny, and functionally exhaust faster than wild‐type littermates.

Under normal biological conditions, LT‐HSCs are primarily responsible for maintaining lifelong hematopoiesis, but are prone to functional exhaustion with age (Olson et al., 2020). To investigate how aging impacts LT‐HSCs, HSPCs, and the hematopoietic system, we compared wild‐type young mice (6–8 weeks) to old mice (18–22 months). As has been published (Lv et al., 2024; Young et al., 2016), old mice significantly accumulated LSKs and LT‐HSCs (Figure 4c). Old mice also had increased ST‐HSCs, but no changes in MPPs (Figure 4c; Figure S4C). Notably, old LT‐HSCs were heavily biased toward MyB‐LT‐HSCs at the expense of LyB‐LT‐HSCs (Figure 4d). To gain insight into the effects of the MyB‐LT‐HSCs bias, we evaluated hematopoiesis downstream. Aged wild‐type mice had increased GMPs and mature myeloid populations and reduced CLPs and B‐ and/or T‐cells in the bone marrow, spleen, and blood (Figure 4e–g; Figure S4C–E). Thus, as age‐driven LT‐HSC functional exhaustion occurs, LyB‐LT‐HSCs deplete, and a significant myeloid bias arises within the hematopoietic compartment. Others have reported through BMT that this myeloid bias is inherent in aged LT‐HSCs (Beerman et al., 2010; Olson et al., 2020). Importantly, our results indicate that *Zranb3*‐deficient LT‐HSCs postsecondary serial BMT displayed an accelerated classic aging/exhausted phenotype.

### 2.5 Aging causes altered DNA replication fork dynamics and DNA damage in HSPCs

A significant contributor to age‐related hematopoietic dysregulation is thought to originate from alterations due to DNA damage and DNA replication stress in HSPCs, especially LT‐HSCs (Flach & Milyavsky, 2018; Li et al., 2016). To ascertain the effects of aging on HSPC DNA damage and replication stress responses, we isolated and evaluated HSPCs from young and old wild‐type mice. Aged LT‐HSCs had increased DNA damage (γH2AX foci; Figure 4h), and no change in DNA breaks (Figure S4F). Fork dynamics showed increased DNA fiber track lengths in old LT‐HSCs (Figure 4i), indicating DNA replication stress is occurring and being bypassed. To determine if these age‐related alterations affected other HSPCs, which have not been well characterized, we evaluated ST‐HSCs and MPPs. Analogous to aged LT‐HSCs, aged ST‐HSCs and MPPs had longer fiber track lengths (Figure 4j, k). However, aged ST‐HSCs did not have increased γH2AX foci, whereas MPPs did, and neither population showed more DNA breaks (Figure 4l; Figure S4F). Therefore, aged ST‐HSCs can clear and prevent accumulation of excess DNA damage whereas LT‐HSCs and MPPs do not. Thus, determining the proteins that protect from and resolve HSPC replication stress and DNA damage on a cell‐type specific basis is critical to understanding HSPC age‐related dysfunction.

### 2.6 Aging increases myeloid bias with Zranb3 loss and reveals a lymphoid bias with Smarcal1 loss

**FIGURE 5:**
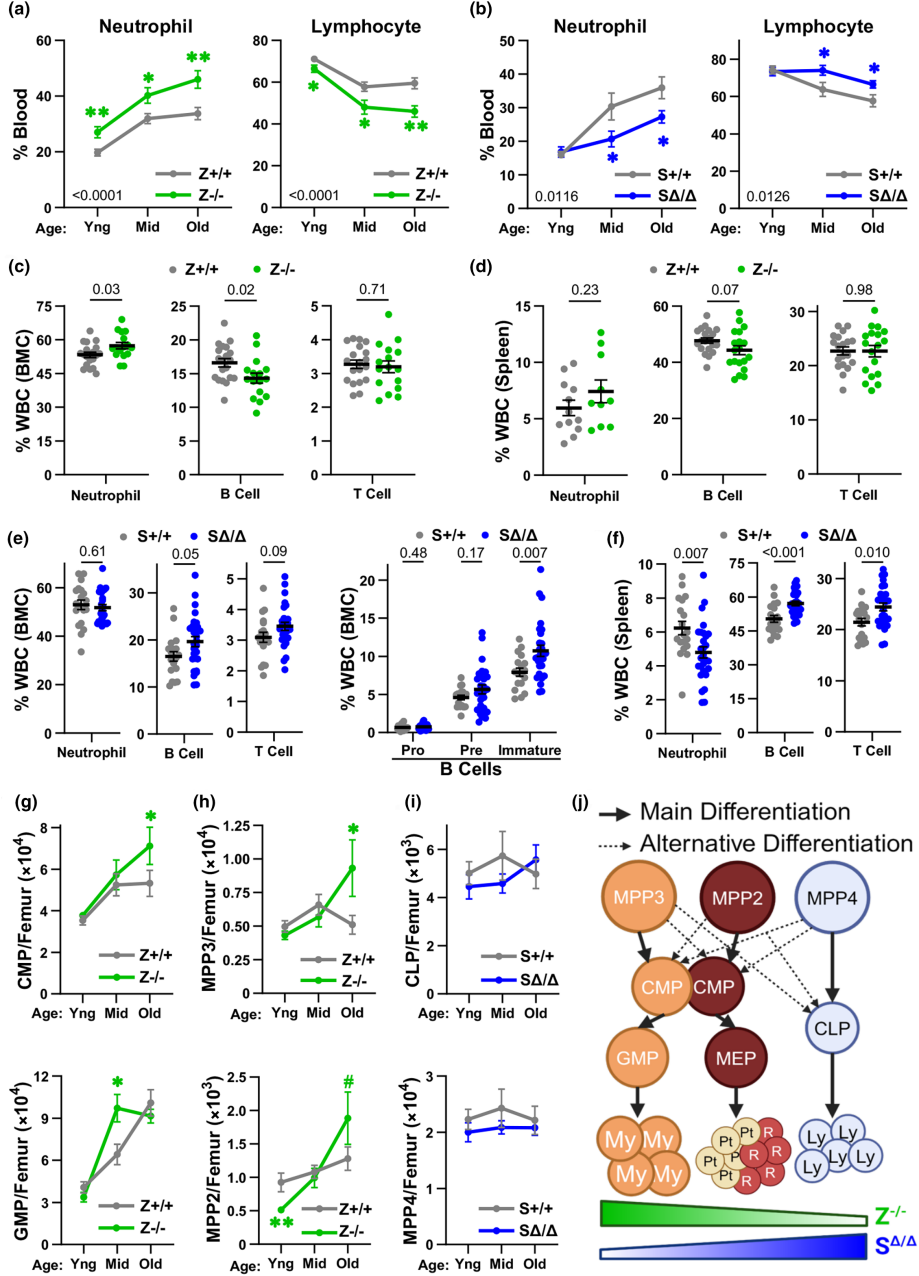
Aging accelerated myeloid‐bias with Zranb3 loss, but lymphoid‐bias emerged with Smarcal1 loss. (a,b) Percentages of neutrophils (left) and lymphocytes (right) in the blood of mice of the indicated genotypes at the indicated age (≥9 mice/genotype/age; mean ± SEM; *p*‐values determined by two‐way ANOVA; *p < 0.05, **p < 0.01 determined by Student's *t*‐test at indicated age). (c–f) Hematopoietic cell populations measured by flow cytometry in the bone marrow (c, e) and spleen (d, f) of old mice of the indicated genotype (≥10 mice/genotype/population; symbols denote individual mice; mean ± SEM; *p*‐values determined by Student's *t*‐tests. (g–i) Numbers of the indicated HSPCs/femur from mice of the indicated genotypes and ages were measured by flow cytometry. At least eight mice/genotype and age; symbols denote individual mice; mean ± SEM; *p*‐values determined by Student's *t*‐tests at the indicated age; #*p* = 0.07, *p ≤ 0.05, **p < 0.01. (j) Graphic depicting the typical differentiation of HSPCs to mature populations (Pietras et al., 2015), and the effects of Zranb3 or Smarcal1 loss (Ly, lymphocyte; My, myeloid cell; Pt, platelet; R, red blood cell).

Given that our data show loss of Zranb3 or Smarcal1 leads to significant replication stress and altered function in HSPCs, and because replication stress, especially in LT‐HSCs, can affect lifelong hematopoiesis, we investigated the roles of Zranb3 and Smarcal1 in physiological hematopoietic aging. Mice deficient in either gene and wild‐type littermate controls at 6–8 weeks (young), 12–15 months (middle age), and 18–22 months (old age) were evaluated. Compared to wild‐type mice, aging *Zranb3*‐deficient mice had progressively increasing percentages of neutrophils and decreasing lymphocytes in their blood (Figure 5a). In stark contrast, aging *Smarcal1*‐deficient mice surprisingly developed decreased neutrophil and increased lymphocyte blood populations (Figure 5b). Bone marrow showed aging *Zranb3*‐deficient mice accumulated neutrophils at the expense of B‐cells, resulting in a strong trend of reduced splenic B‐cells (Figure 5c, d; Figure S5A–D). Conversely, aging *Smarcal1*‐deficient mice resulted in progressive increases in bone marrow and spleen lymphocyte populations, particularly bone marrow immature B‐cells, IgD^+^IgM^+^ splenic B cells, and CD8^+^ T cells, leading to decreases in splenic myeloid cells (Figure 5e, f; Figure S5E‐H).

To gain insight into the cause of the differences in the periphery, we evaluated lineage progenitors in the bone marrow of aged *Zranb3*‐ or *Smarcal1*‐deficient mice. Accompanying the increase in peripheral myeloid populations, aged *Zranb3* ^−/−^ mice also accumulated myeloid progenitors (CMPs and GMPs) earlier than wild‐type mice (Figure 5g). Additionally, old *Zranb3* ^−/−^ mice had increased myeloid‐biased MPP3s and MPP2s (Figure 5h), which are biased toward erythroid/megakaryocytic and myeloid lineages (Pietras et al., 2015). There were no differences, compared to wild‐type mice, in lymphoid‐biased MPP4s in aging *Zranb3* ^−/−^ mice (Figure S5I). In aged *Smarcal1* ^*Δ/Δ*^ mice, despite a peripheral lymphoid bias, there were no significant differences in CLPs or lymphoid‐biased MPP4s (Figure 5i). Although young *Smarcal1‐*deficient mice had decreased CMPs, after aging, myeloid progenitor (CMP, GMP, MPP2, and MPP3) numbers were not altered compared to wild‐type controls (Figure S5J). However, old *Smarcal1* ^*Δ/Δ*^ mice had reduced myeloid populations (Figure 5f) and MEPs (Figure S5J) suggesting a problem during myelopoiesis. Thus, with aging, Zranb3 loss results in a distinct and progressive myeloid‐bias beyond that in wild‐type mice, but Smarcal1 loss results in a progressive lymphoid bias (Figure 5j), further distinguishing the protective functions of Zranb3 and Smarcal1 in hematopoiesis.

Of note, HSPCs in either *Zranb3* ^+/−^ or *Smarcal1* ^+/Δ^ mice displayed a haploinsufficiency effect. For example, *Zranb3* ^+/−^ mice also had increased neutrophils in the blood, and *Smarcal1* ^+/Δ^ mice had increased lymphocytes in the blood (Figure S5K,L) compared to wild‐type mice. Also, many, but not all, populations within the bone marrow and spleen showed intermediate phenotypes with heterozygosity during aging (Figure S5M–V). Thus, the effects of *Zranb3* and *Smarcal1* deficiency may be gene dosage‐dependent.

### 2.7 Zranb3 and Smarcal1 uniquely mitigate HSPC replication stress in old age

**FIGURE 6:**
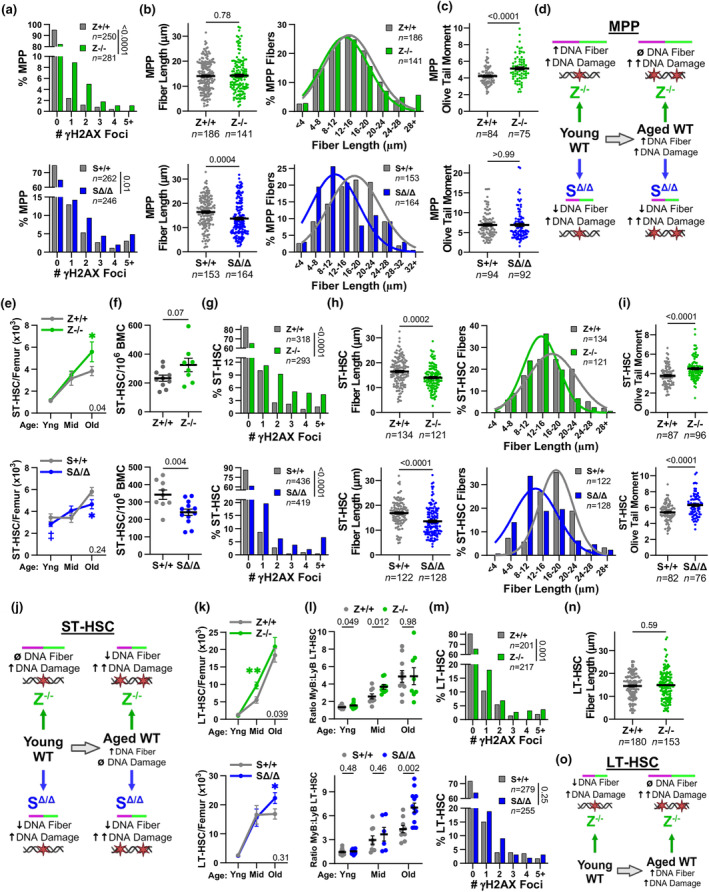
Zranb3 and Smarcal1 independently mitigate DNA replication stress in HSPCs. (a–c) Flow cytometry isolated MPPs from old mice (genotype indicated). Percentage of MPPs with the indicated γH2AX foci/cell (a, immunofluorescence). DNA fiber lengths (b left) and binned fiber lengths (b right) in isolated MPPs. Comet assay Olive tail moments from MPPs (c). (d) Schematic summarizing results in young and old MPPs. (e) ST‐HSCs/femur of the indicated genotypes and ages. (f) ST‐HSCs/10^6^ BMCs from old mice (genotype indicated) (8–13 mice/genotype; symbols are individual mice; mean ± SEM; *p*‐values determined by Student's *t*‐tests). (g–i) Flow cytometry isolated ST‐HSCs from old mice (genotype indicated). Percentage of ST‐HSCs with the indicated γH2AX foci/cell (g, immunofluorescence). DNA fiber lengths (h left) and binned fiber lengths (h right) in isolated ST‐HSCs. Comet assay Olive tail moments from ST‐HSCs (i). (j) Schematic summarizing results in young and old ST‐HSCs. (k) LT‐HSCs/femur of the indicated genotypes and ages. (l) Ratio of MyB‐LT‐HSCs to LyB‐LT‐HSCs of the indicated genotypes and ages as measured by flow cytometry (≥6 mice/genotype/age; symbols are individual mice; mean ± SEM; *p*‐values determined by Student's *t*‐test). (m, n) Flow cytometry isolated LT‐HSCs from old mice (genotypes indicated). Percentage of LT‐HSCs with the indicated γH2AX foci/cell (m, immunofluorescence). DNA fiber lengths in isolated LT‐HSCs (n). (o) Schematic summarizing the results in young and old LT‐HSCs. For (a, g, and m) “*n*” denotes numbers of cells; *p*‐values determined by chi‐squared test for trend. For (b, h, and n) “*n*” is number of fibers; symbols are individual fibers; mean ± SEM; *p*‐values determined by Student's *t*‐test; curves are calculated Gaussian distribution. For c and i, “*n*” is number of cells; symbols are individual cells; mean ± SEM; *p*‐value determined by Student's *t*‐tests. Representative experiments in (a–c, g–i, m, and n). For e and k, mean ± SEM; ≥8 mice/genotype/age; *p*‐values within graphs determined by two‐way ANOVA; *p*‐values determined by *t*‐test at specific ages are ‡*p* = 0.06, *p < 0.05, **p < 0.01.

To understand the contributions of Zranb3 and Smarcal1 to mitigating age‐related DNA replication stress, we evaluated old HSPCs. Similar to young MPPs, loss of either protein in old MPPs caused increased DNA damage (γH2AX foci; Figure 6a). In young MPPs, fork dynamics were altered without Zranb3 or Smarcal1 (Figure 2c, g). Aging of wild‐type MPPs also caused DNA replication stress that led to increased fiber lengths (Figure 4k), and while Zranb3 loss in old age did not further exacerbate alterations at replication forks of MPPs (Figure 6b), there were increased DNA breaks (Figure 6c). This suggests that in MPPs, aging without Zranb3 engaged a DNA replication stress response mechanism that bypassed the stress but compromised DNA stability. Conversely, old *Smarcal1* ^*Δ/Δ*^ MPPs had reduced fiber lengths without more DNA breaks (Figure 6b, c), indicating fork stalling provided opportunities for DNA repair. Thus, similar to young MPPs, both Zranb3 and Smarcal1 are uniquely essential to mitigate DNA replication stress and DNA damage in aged MPPs (Figure 6d).

In young ST‐HSCs, while loss of either gene increased DNA damage and resulted in trends toward decreased cell numbers (Figure 2j, l, m, p), only Smarcal1 loss led to DNA fork stalling (Figure 2n). Remarkably, old *Zranb3* ^−/−^ mice accumulated ST‐HSCs (Figure 6e, f), whereas old *Smarcal1* ^Δ/Δ^ mice had reduced ST‐HSC numbers (Figure 6e) and frequency (Figure 6f). Additionally, both genotypes of aged ST‐HSCs showed increased DNA damage (γH2AX foci), replication fork stalling (decreased DNA fiber length), and DNA breaks (Figure 6g–i). Thus, aging in ST‐HSCs causes increased reliance on both Zranb3 and Smarcal1 to mitigate the significant DNA replication stress caused during aging hematopoiesis (Figure 6j).

Evaluation of aging LT‐HSCs revealed *Zranb3* ^−/−^ mice exhibited classic hallmarks of accelerated aging: accumulation of more LT‐HSCs and increased MyB‐LT‐HSCs earlier in life compared to wild‐type littermates (Figure 6k, l; Figure S6A), and more γH2AX foci (Figure 6m). Although young *Zranb3*‐deficient LT‐HSCs had DNA fork stalling (Figure 2q), aged *Zranb3*‐deficient LT‐HSCs had no further increase in DNA replication stress beyond that induced by aging (Figure 6n, o; Figure S6B), or changes in DNA breaks (Figure S6C). In contrast, LT‐HSCs from young and middle age *Smarcal1*‐deficient mice were unaffected (Figure 6k, l; Figure S6A). LT‐HSCs and MyB‐LT‐HSCs were only increased at old age in mice lacking Smarcal1 (Figure 6k, l), potentially compensating for diminished ST‐HSCs. Old *Smarcal1* ^Δ/Δ^ LT‐HSCs also showed no increase in DNA damage (γH2AX foci; Figure 6m) or breaks (Figure S6C). Taken together, the hematopoietic alterations during aging indicate Zranb3 loss accelerates hematopoietic aging and LT‐HSC dysfunction, whereas effects from Smarcal1 loss are primarily observed in downstream progenitors, such as ST‐HSCs, and lineage‐specific cells.

### 2.8 Compounded replication stress response defects in DKO mice

Our results indicate that despite Zranb3 and Smarcal1 having similar DNA fork reversal functions in the replication stress response (Betous et al., 2012; Ciccia et al., 2012; Poole & Cortez, 2017), they have unique, nonredundant roles in HSPCs and during aging hematopoiesis. To determine the effects of losing both proteins on hematopoiesis, *Zranb3* was deleted on the *Smarcal1* ^*Δ/Δ*^ background, generating double knockout (DKO) mice. Loss of both genes, surprisingly, did not cause embryonic lethality or alter the expected Mendelian ratios of those born (Figure S7A), suggesting the recently identified alternative mechanisms for mitigating DNA replication stress are active during embryogenesis. Evaluation of young (6–8 weeks old) DKO mice showed mild differences in HSPCs, trending toward reduced MPP2s and MPP3s beyond Smarcal1 loss alone (Figure S7B), similar, but more muted, to the phenotype without Zranb3. In mice without Zranb3 or Smarcal1 alone, HSPC alterations were not due to changes in the maintenance of hematopoietic cells by the bone marrow niche (Figure S3B–G), but it is possible that the niche microenvironment may be impacted in DKO mice.

**FIGURE 7:**
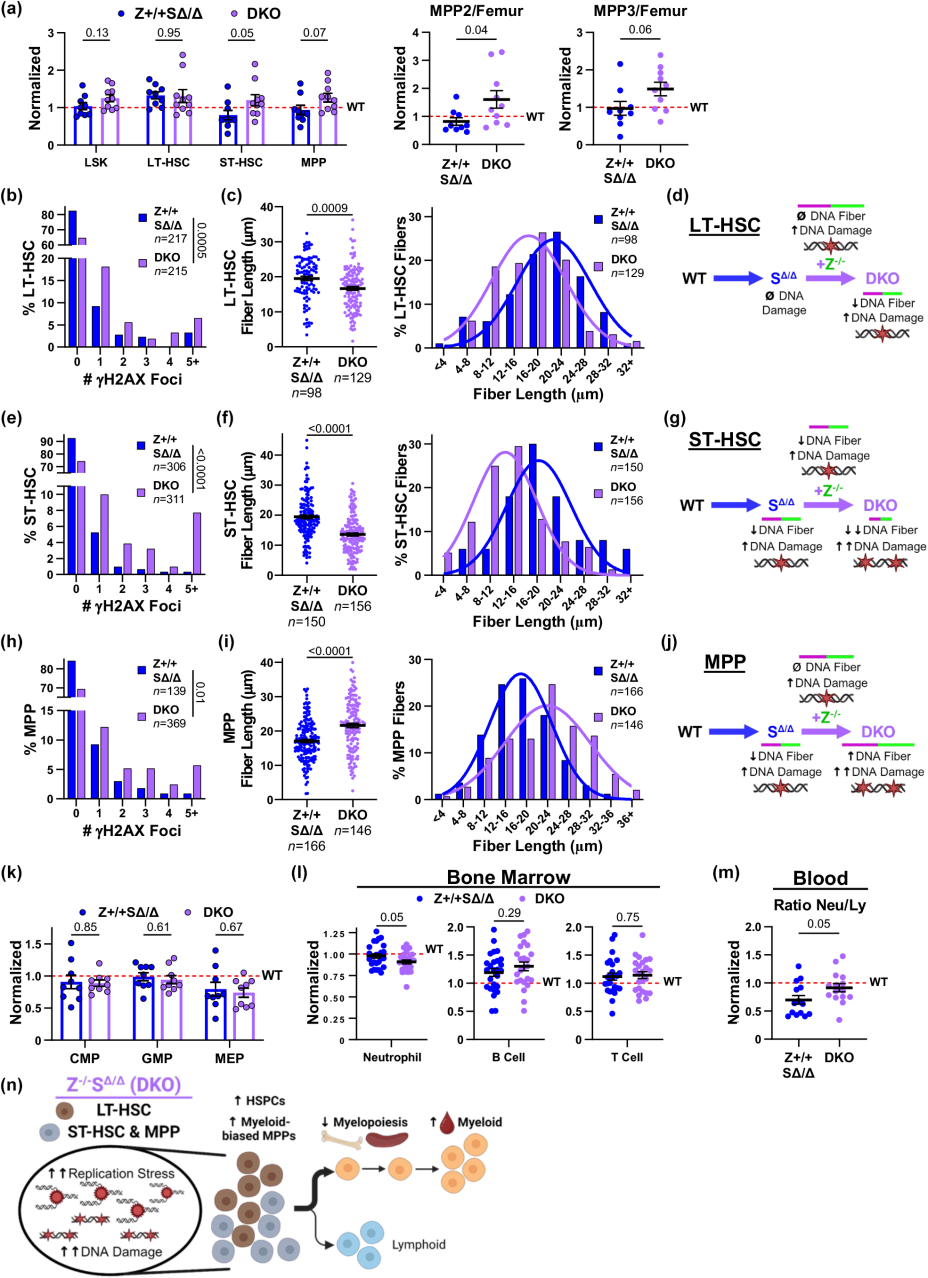
Simultaneous loss of Zranb3 and Smarcal1 have compounded effects on DNA replication stress and hematopoiesis. (a) Normalized values of numbers of specific HSPCs/femur from old mice of the indicated genotypes. (b, c) Flow cytometry isolated LT‐HSCs from old mice of the indicated genotypes. Percentage of LT‐HSCs with the indicated γH2AX foci/cell (b, immunofluorescence). DNA fiber lengths (c left) and binned fiber lengths (c right) in isolated LT‐HSCs. (d) Schematic summarizing the results in old LT‐HSCs. (e, f) Flow cytometry isolated ST‐HSCs from old mice of the indicated genotypes. Percentage of LT‐HSCs with the indicated γH2AX foci/cell (e, immunofluorescence). DNA fiber lengths (f left) and binned fiber lengths (f right) in isolated ST‐HSCs. (g) Schematic summarizing the results in old ST‐HSCs. (h, i) Flow cytometry isolated MPPs from old mice of the indicated genotypes. Percentage of MPPs with the indicated γH2AX foci/cell (h, immunofluorescence). DNA fiber lengths (i left) and binned fiber lengths (i right) in isolated MPPs. (j) Schematic summarizing the results in old MPPs. (k) Normalized numbers of specific HSPCs/femur from old mice of the indicated genotypes. (l) Normalized percentages of hematopoietic cells from the bone marrow of old mice of the indicated genotypes. (m) Normalized ratios of neutrophils to lymphocytes in the blood of old mice of the indicated genotypes. (n) Graphical summary of the effects of simultaneous loss of Zranb3 and Smarcal1 on replication stress in HSPCs and downstream hematopoietic populations after aging. For (a and k–m) data normalized to wild‐type values of each and is set at one (dotted line); ≥7 mice/genotype/age; symbols denote individual mice; mean ± SEM; *p*‐values determined by Student's *t*‐tests. For (b, e, and h) “*n*” denotes numbers of cells; *p*‐values determined by chi‐squared test for trend. For (c, f, and i), “*n*” is number of fibers; symbols denote individual fibers; mean ± SEM; *p*‐values determined by Student's *t*‐test; curves are calculated Gaussian distribution. Representative experiments shown in (b, c, e, f, h, i).

Because lack of either gene has diverse effects on aging HSPCs and hematopoiesis, DKO mice and *Smarcal1* ^Δ/Δ^ littermates were aged to 18–22 months (old) and evaluated. After aging, Smarcal1 loss alone resulted in reduced ST‐HSCs and no changes to MPPs (Figures 5i and 6e, f; Figure S5J). Additional loss of Zranb3 in DKO mice resulted in significantly increased ST‐HSCs and MPP2s, and a strong trend up for MPP3s and total MPPs, but not MPP4s (Figure 7a; Figure S7C), which reflects the differences in old mice after Zranb3 loss alone.

To determine whether simultaneous loss of both proteins affects DNA damage and replication stress responses, we isolated HSPCs from old mice. While Smarcal1 loss in LT‐HSCs did not increase DNA damage (Figure 6m), additional loss of Zranb3 (DKO) caused increased γH2AX foci (Figure 7b), analogous to *Zranb3*‐deficiency alone (Figure 6m). Furthermore, DKO LT‐HSCs exhibited increased replication fork stalling (reduced fiber lengths; Figure 7c), indicating loss of both proteins in old LT‐HSCs is more detrimental than Smarcal1 loss alone (Figure 7d). Similarly, whereas *Smarcal1* ^*Δ/Δ*^ ST‐HSCs displayed increased DNA damage and fork stalling (Figure 6g, h), DKO ST‐HSCs had amplified levels of DNA damage (γH2AX foci) and fork stalling (Figure 7e–g), indicating compounded replication stress. Therefore, Zranb3 and Smarcal1 act cooperatively to prevent DNA replication stress and DNA damage in old LT‐ and ST‐HSCs.

Evaluations of old DKO MPPs revealed further increases in γH2AX foci and replication stress beyond *Smarcal1*‐deficiency alone (Figure 7h–j). In contrast to LT‐ and ST‐HSCs, however, DKO led to increased fiber track lengths compared to *Smarcal1* ^*Δ/Δ*^ littermates (Figure 7i). Furthermore, DKO MPPs also had increased DNA breaks (Figure S7D). Thus, in old MPPs specifically, additional replication stress from simultaneous Zranb3 and Smarcal1 loss activates an alternative mechanism to bypass the stress that results in DNA damage and breaks. Altogether, the DNA replication stress response in old HSPCs is further impaired without both Zranb3 and Smarcal1.

### 2.9 Simultaneous loss of Zranb3 and Smarcal1 impedes hematopoietic lineage development

Since the LSK populations from DKO mice appeared impacted, we investigated the production of downstream hematopoietic populations. In young DKO mice, both CMP and GMP levels were similar to *Smarcal1* ^Δ/Δ^ mice (Figure S7E), which were reduced compared to wild‐type mice (Figure S5J). CLPs and mature hematopoietic populations in the bone marrow, spleen, and blood were largely unaffected in DKO mice (Figure S7E‐H). Thus, oligopotent lineage progenitors from young, unstressed DKO mice display *Smarcal1* ^Δ/Δ^‐like phenotypes.

After aging, Zranb3 loss led to a dramatic myeloid bias, whereas aged *Smarcal1*‐deficient mice displayed a lymphoid bias (Figure 5). Remarkably, despite accumulated myeloid‐biased MPP2s and MPP3s (Figure 7a), old DKO mice had no increase in CMPs, GMPs, or MEPs compared to *Smarcal1* ^Δ/Δ^ littermates (Figure 7k). Moreover, old DKO mice had reduced mature myeloid bone marrow populations compared to old *Smarcal1* ^*Δ/Δ*^ mice (Figure 7l; Figure S7I). CLPs were unchanged (Figure S7J), but compared to old wild‐type mice, old DKO mice retained elevated levels of bone marrow and spleen B and T cells, analogous to those in old *Smarcal1*‐deficient mice (Figure 7l; Figure S7I,K). Also, spleen myeloid populations remained diminished, suggesting myelopoiesis was impacted in DKO mice (Figure S7K). However, the blood of old DKO mice had an increased neutrophil to lymphocyte ratio compared to *Smarcal1* ^Δ/Δ^ littermates (Figure 7m), suggesting an increased push to produce myeloid cells with Zranb3 loss. While we demonstrated that mice lacking Zranb3 or Smarcal1 have HSPC cell intrinsic hematopoiesis effects (Figures 1 and 3, Figure 3b–g), and the same is expected for DKO, we cannot exclude a consequence on hematopoiesis with loss of both from the bone marrow microenvironment. Old *Zranb3* ^+/‐^ *Smarcal1* ^Δ/Δ^ mice largely showed intermediate phenotypes, suggesting these phenotypes also occurred in a gene dose‐dependent manner (Figure S7L–O). Thus, in old DKO mice, while Zranb3 loss biases hematopoietic output of early progenitors toward increased myeloid populations, Smarcal1 loss appears to significantly impede the differentiation of those myeloid cells (Figure 7n). Taken together, both Zranb3 and Smarcal1 have unique and critical roles in HSPCs that cooperatively protect hematopoiesis.

## 3 DISCUSSION

**FIGURE 8:**
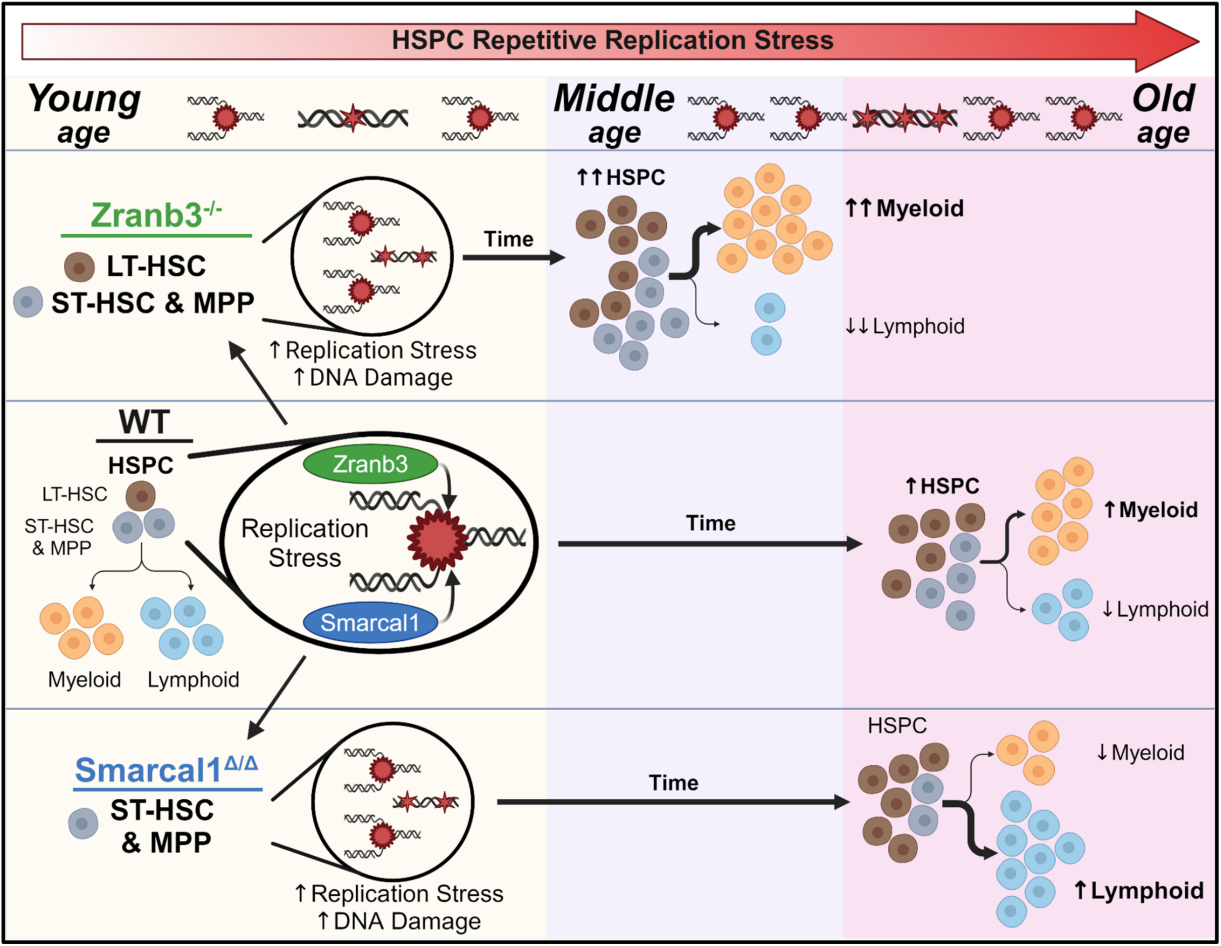
Aging hematopoiesis is profoundly dysregulated in distinct ways with loss of Zranb3 or Smarcal1‐mediated DNA replication stress response. Schematic of the roles of Zranb3 and Smarcal1 in uniquely mitigating DNA replication stress and damage in HSPCs and maintaining populations during lifelong hematopoiesis. Repetitive DNA replication stress in wild‐type HSPCs over a lifetime leads to LT‐HSC functional exhaustion and myeloid bias. Zranb3 loss causes increased DNA replication stress and damage in both LT‐HSCs and progenitors, leading to an accelerated aging phenotype including earlier accumulation of LT‐HSCs and myeloid‐bias. Smarcal1 loss primarily impacts hematopoietic progenitor populations, leading to a lymphoid bias.

During lifelong hematopoiesis, HSPCs need to repetitively replicate to maintain hematopoietic homeostasis and react to hematopoietic system insults (Flach & Milyavsky, 2018; Olson et al., 2020; Rossi et al., 2008). Yet, little is understood about HSPC responses to physiological DNA replication stress, the proteins required to maintain DNA replication fidelity, and aging‐induced alterations or their effects. Here we report that two evolutionarily conserved and closely related DNA replication fork remodeling enzymes, Zranb3 and Smarcal1, are required to respond to HSPC DNA replication stress, protect replication fork function, and maintain DNA integrity during hematopoiesis, especially during aging. Importantly, although Zranb3 and Smarcal1 have similar fork reversal biochemical functions (Betous et al., 2012; Ciccia et al., 2012; Poole & Cortez, 2017) and ubiquitous expression in HSPCs (Heng et al., 2008), our unprecedented study surprisingly reveals nonredundant, HSPC‐specific biological functions. Our results bring to light the unique biological and biochemical needs of specific HSPCs under physiological replication stresses. Notably, Zranb3 has essential roles in LT‐HSCs, and its loss caused accelerated myeloid‐biased hematopoietic aging, whereas Smarcal1 is critical for downstream HSPCs (Figure 8). Overall, our results reveal hematopoiesis relies on both Zranb3 and Smarcal1 to mitigate DNA replication stress, thus averting DNA damage, breaks, and replication failure that cause hematopoietic cell deficiencies during intrinsic physiological HSPC stress and aging.

Although HSPCs experience DNA replication stress from organismal development and normal hematopoiesis, aging causes additional DNA replication stress and DNA damage in HSPCs (Flach & Milyavsky, 2018; Li et al., 2016; Rossi et al., 2007; Rossi et al., 2008). Consequently, as we here and others have shown, aging leads to pronounced alterations in hematopoiesis, including accumulation of functionally exhausted LT‐HSCs, distinct myeloid cell bias (Flach & Milyavsky, 2018; Lv et al., 2024; Rossi et al., 2008) (Figure 8), and increased risk of hematopoietic malignancy (Olson et al., 2020; Rossi et al., 2008). It is thought that aging‐induced hematopoietic abnormalities are due to accrual and consequences of DNA damage, marked by persistent γH2AX foci (Beerman et al., 2014; Flach et al., 2014; Rossi et al., 2007; Rube et al., 2011), from a lifetime of repeated DNA replication stress induced insults in LT‐HSCs during hematopoiesis (Flach & Milyavsky, 2018; Li et al., 2016; Olson et al., 2020). This drives LT‐HSC functional exhaustion (Beerman et al., 2014; Flach et al., 2014; Flach & Milyavsky, 2018; Walter et al., 2015), which likely pushes ST‐HSCs and MPPs to compensate, causing the increased DNA replication stress we observed. Here we show that Zranb3 loss in young LT‐HSCs, ST‐HSCs, and MPPs causes DNA replication stress, damage, and/or breaks, which led to reduced numbers of healthy LyB‐LT‐HSCs and MPPs. With chronic DNA replication stress in LT‐HSCs, aged *Zranb3* ^−/−^ mice accumulated excess HSPC DNA damage with stalled replication forks and DNA breaks now detectable in ST‐HSCs. There were also obvious signs of accelerated hematopoietic aging in *Zranb3*‐deficient mice, including progressive expansion of myeloid lineages and reductions of lymphoid populations, and, pointedly, an accumulation of MyB‐LT‐HSCs already evident in 1‐year‐old mice (Figure 8). Thus, Zranb3 is essential to mitigate DNA replication stress in LT‐HSCs, ST‐HSCs, and MPPs during normal and aged hematopoiesis, and potentially also protects downstream populations.

Previous investigations in zebrafish reported Smarcal1 loss did not affect LSK populations (Huang et al., 2010). Our study in mice; however, establishes a vital role for Smarcal1 in mammalian HSPCs during hematopoiesis induced DNA replication stress. In young and aged mice, Smarcal1 loss did not affect LT‐HSCs, but detrimentally impacted the DNA replication stress responses in ST‐HSCs and MPPs, resulting in DNA damage in both and DNA breaks in ST‐HSCs. Notably, antithetical to typical aging and starkly juxtaposed to aged *Zranb3*‐deficient mice, aging without Smarcal1 caused reduced ST‐HSCs, CMPs, MEPs, and neutrophils, and increased lymphocytes, suggesting diminished myelopoiesis and/or diversion toward lymphopoiesis (Figure 8). Therefore, both Zranb3 and Smarcal1 have integral, and distinctly unique, functions in the maintenance of HSPCs, including cell‐type‐specific contributions.

Biological evidence further demonstrating Zranb3 and Smarcal1 cell type preferential contributions emerged from the BMT analyses. Limiting dilution BMT, which tests the frequency of functional LT‐HSCs (Purton & Scadden, 2007), and serial BMTs, which tests LT‐HSC repopulation ability (Harrison & Astle, 1982; Purton & Scadden, 2007), both showed that Zranb3, and not Smarcal1, was essential for maintaining functional LT‐HSCs. The reduced reconstitution from BMCs lacking Smarcal1 upon competitive BMT was likely due to reduced function and DNA replication stress response of ST‐HSCs, MPPs, and possibly other progenitor cells. Moreover, the increase in functional *Smarcal1*‐deficient LT‐HSCs detected by limiting dilution BMT are likely a consequence of these ST‐HSC defects, as compensation for reduced hematopoietic output, thus ensuring hematopoiesis and bone marrow reconstitution. Additionally, these data explain why more hematopoietic defects were not observed in young *Smarcal1*‐deficient mice, and why there were increased LT‐HSCs in old age mice but decreased ST‐HSCs. In contrast, reduced functional LT‐HSCs and defects in lineage differentiation in young *Zranb3*‐deficient mice explain the diminished reconstitution after competitive and serial BMT, and accelerated myeloid bias and LT‐HSC functional exhaustion after BMT or aging. Thus, Zranb3 protects the function of LT‐HSCs and likely downstream progenitors, whereas Smarcal1 has preferential roles in maintaining ST‐HSCs and other hematopoietic progenitors (Figure 8).

Evaluation of HSPCs in mice lacking both Zranb3 and Smarcal1 provided further insight into their HSPC specific and nonredundant functions. Notably, there were compounded effects in DKO mice that could be traced back to either loss of Zranb3, Smarcal1, or both. For example, similar to Zranb3 loss alone, old DKO mice showed myeloid‐biased hematopoietic cell production, although lineage committed cells undergoing myelopoiesis and/or lymphopoiesis were impacted, similar to old *Smarcal1* ^Δ/Δ^ mice. Importantly, our data show Zranb3 and Smarcal1 act cooperatively to mitigate DNA replication stress, as old DKO ST‐HSCs and MPPs had increased DNA replication stress and DNA damage compared to old *Smarcal1* ^Δ/Δ^ littermates. However, while Zranb3 or Smarcal1 loss alone did not affect the bone marrow niche, we cannot exclude the possibility that simultaneous loss of both impacted the bone marrow microenvironment. Nevertheless, these data highlight the profoundly different ways that Zranb3 and Smarcal1 protect hematopoiesis.

The evolutionarily conserved mechanisms that ensure high fidelity DNA replication and response to DNA replication stress have been emerging in recent years (Flach & Milyavsky, 2018; Saxena & Zou, 2022), but the exact biological circumstances necessitating each remains unresolved. Initially, biochemical studies showed that DNA replication stress activated fork reversal by Zranb3 or Smarcal1 to stabilize the replication fork until DNA was repaired (Betous et al., 2012; Ciccia et al., 2012; Poole & Cortez, 2017). We reported that both Zranb3 and Smarcal1 are required to mitigate chronic oncogene‐induced DNA replication stress in primary pre‐B cells (Puccetti et al., 2019). However, recently, we reported that circumstances necessitating urgent DNA replication, such as acute responses to a mock viral infection (pI:pC injection), favored DNA fork repriming by PrimPol over fork reversal by Zranb3 or Smarcal1 (Jacobs et al., 2022). In our current study, we unexpectedly determined the same physiological HSPC stresses, including aging, resulted in population‐specific utilization of Zranb3 and/or Smarcal1 catalyzed fork reversal or an alternative mechanism. For example, longer fiber track lengths, indicating activation of repriming or possibly, new origin firing to bypass problematic DNA forks, were evident in young *Zranb3* ^−/−^ MPPs, but young *Smarcal1* ^Δ/Δ^ MPPs had reduced fiber track lengths caused by fork reversal and stalled forks. In old ST‐HSCs lacking Zranb3 and/or Smarcal1, however, only fork stalling occurred. Thus, our data provide unprecedented physiologically‐relevant biological evidence that even though LT‐HSCs, ST‐HSCs, and MPPs are all undifferentiated hematopoietic cells, they uniquely respond to DNA replication stress, depending on expression of response proteins, the speed at which DNA replication is needed, and other factors, such as aging, that could confer additional challenges to mitigating DNA replication stress.

Overall, our data reveal critical, physiological insight into the complex mechanisms that respond to DNA replication stress during normal and aging hematopoiesis, by revealing that the ubiquitous and evolutionarily conserved Zranb3 and Smarcal1 have unique roles in protecting LT‐HSC and/or HSPC proliferation. Our results also support the new concept that the DNA replication stress response activated in HSPCs has plasticity and is dependent on the specific conditions. Importantly, in humans, repetitive or increased HSPC DNA replicative stress, such as from aging or BMT, may lead to mutations that cause or predispose to clonal hematopoiesis and/or the development of hematologic malignancies (Olson et al., 2020; Wong et al., 2018). Furthermore, our results suggest performing therapeutic BMTs with HSCs from individuals with reduced Zranb3 or Smarcal1 function could result in adverse consequences, including graft failure, hematopoietic cell defects, or malignant transformation. Conversely, since these and our previous data (Puccetti et al., 2017; 2019) demonstrate that loss of function of Zranb3 or Smarcal1 can negatively impact hematopoietic cell DNA replication, targeting of either may provide novel avenues of therapeutic intervention to treat aggressive hematopoietic malignancies and/or hyperproliferative hematologic conditions/syndromes.

## 4 MATERIALS AND METHODS

### 4.1 Mice

Congenic CD45.2 C57Bl/6 *Zranb3* ^+/−^ and *Smarcal1* ^+/Δ^ mice were generated from mice purchased from the Texas Institute of Genomic Medicine or previously provided by Dr. Cornelius Boerkoel, University of British Columbia (Baradaran‐Heravi et al., 2012), respectively. Mice lacking both genes and *Smarcal1* ^Δ/Δ^ knockout littermate controls were generated by breeding. Mice 6–8 weeks old were young, 12–15 months old were middle‐aged, and 18–22 months old were old mice. Mice and tissues were excluded if pathologies significantly impacting hematopoiesis/hematopoietic cells were evident or for specific experimental approaches, conditions were met with statistical analyses/outlier tests within genotypes (indicated in Statistics section below). CD45.1 (B6.SJL‐Ptprc^a^ Pepc^b^/BoyJ) mice were purchased from Jackson labs. All experiments included male and female littermate‐matched mice, were approved by the Thomas Jefferson University Institutional Animal Care and Use Committee, and adhered to all state and federal guidelines.

### 4.2 Competitive, serial, and limiting dilution bone marrow transplantation

After lysing red blood cells (RBCs) (Gey's solution), BMCs from leg bones of CD45.2^+^ wild‐type and littermate‐matched *Zranb3* ^*−/−*^ or *Smarcal1* ^*Δ/Δ*^ mice were mixed in ratios described below and transplanted (retro‐orbital injection) into lethally‐irradiated CD45.1^+^ wild‐type recipients. Blood was assessed monthly, and after at least 16 weeks, other tissues were evaluated as described below. For competitive limiting dilution transplant, CD45.1^+^ wild‐type BMCs (300,000) were mixed with either 3000 (1:100; 8–9 recipients/transplant), 10,000 (1:30; 5–7 recipients/transplant), 30,000 (1:10; 5–11 recipients/transplant) or 100,000 (1:3 ratio; 4–5 recipients/transplant) CD45.2^+^ wild‐type or littermate‐matched *Zranb3* ^*−/−*^ or *Smarcal1* ^*Δ/Δ*^ BMCs (pool of 2 mice/genotype/transplant); excess donor mixtures were analyzed by flow cytometry as described below, and matched expected results (data not shown). For serial transplants, 1 × 10^6^ BMCs (after RBC lysis) from CD45.2^+^ *Zranb3* ^*−/−*^ or *Smarcal1* ^*Δ/Δ*^ mice or littermate‐matched wild‐type controls were transplanted into CD45.1^+^ recipients. Following 16 weeks recovery, secondary transplants were performed by transplanting 1 × 10^6^ BMCs into new recipients, and tertiary transplants were similarly performed 16 weeks postsecondary transplant. At least 16 weeks after secondary and tertiary transplantation, mice were euthanized and evaluated, as described below. LT‐HSC subtype ratios were only determined in mice with at least 20 CD45.2^+^ LT‐HSC events. Chimeric ratios were determined in recipients with at least 7% total CD45.2^+^ bone marrow reconstitution.

### 4.3 Blood counts

Blood from mice was collected by submandibular bleed into tubes with K3‐EDTA (MiniCollect). Samples were analyzed using a GENESIS Veterinary Hematology Analyzer in the Translational Research and Pathology core facility.

### 4.4 HSC homing assay

RBC lysed BMCs (10 × 10^6^) from CD45.2^+^ wild‐type or littermate‐matched *Zranb3* ^*−/−*^ or *Smarcal1* ^*Δ/Δ*^ were transplanted into lethally‐irradiated recipients (10Gy) after incubating them in CFSE (1:1000) for 20 min at 37°C. The number of CFSE^+^ HSCs transplanted were determined by flow cytometry at time of transplantation. Bone marrow was harvested 24 h posttransplantation by crushing bones (femur, tibia, and pelvis) and analyzed by flow cytometry. The percentage of homed HSCs was determined by calculating the amount of CFSE^+^ HSCs in the bone marrow divided by the amount of CFSE^+^ HSCs transplanted.

### 4.5 Bone marrow niche test

RBC lysed BMCs (1 × 10^6^) from CD45.1^+^ wild‐type mice were transplanted into lethally‐irradiated (10Gy) littermate‐matched CD45.2^+^ wild‐type and *Zranb3* ^*−/−*^ or *Smarcal1* ^*Δ/Δ*^ mice. Mice were followed for 16 weeks and hematopoietic populations were evaluated by flow cytometry as described below.

### 4.6 Flow cytometric analysis of blood, spleen, and bone marrow

*Blood leukocyte analysis*: Blood was obtained by submandibular bleeding into tubes containing 10% EDTA (disodium). RBCs were lysed (Gey's solution), PBS added, and samples centrifuged. *Spleen and bone marrow analysis*: Spleen and bone marrow were harvested from mice and single‐cell suspensions were generated. Bone marrow from BMT recipient mice, RBCs were lysed, PBS added, and samples centrifuged. Immunophenotyping was performed as previously described (Puccetti et al., 2017). *HSPC analysis*: Bone marrow was flushed into FACS buffer (PBS, 1% FBS). Cells were centrifuged and RBCs were lysed (Gey's solution). Samples were incubated overnight (4°C) in a mixture of antibodies. Cells were then incubated with streptavidin‐linked Pacific‐Blue for 30 min at 4°C in FACS buffer. FACS buffer was added, centrifuged, and samples analyzed by flow cytometry. Samples were either run live or fixed with either BD Cytofix/Cytoperm Buffer alone or in combination with BD Cytoperm Permeabilization Buffer Plus. *All flow assays*: Antibodies are listed in Table S2. Samples were evaluated using BD LSRII, BD Fortessa, BD Celesta, or BD Symphony (BD‐Biosciences) cytometers and data analyzed using FlowJo software.

### 4.7 HSPC isolation

Bone marrow was harvested by crushing bones and RBCs lysed (Gey's solution). cKit enrichment was performed using the Miltenyi MACS Cell Separation protocol and CD117 MicroBeads on either LS Columns or the AutoMACS system. Cells were then incubated with B220‐Biotin, CD3‐Biotin, Gr1‐Biotin, CD11b‐Biotin, Ter119‐Biotin, CD19‐Biotin, CD4‐Biotin, CD8‐Biotin, either Sca1‐FITC or Sca1‐BUV395, CD150‐PECy7, cKit‐APC, and CD48‐APCCy7 in FACS buffer for 45 min on ice. Samples were washed with FACS buffer then incubated with streptavidin linked‐Pacific‐Blue for 30 min at 4°C. After washing with FACS buffer, cells were sorted using a BD AriaII (BD‐Biosciences).

### 4.8 γH2AX immunofluorescence

Immunofluorescent detection of γH2AX foci was performed on blinded samples as previously described (Puccetti et al., 2017). Images were captured and analyzed on Nikon C2 or A1R laser scanning confocal microscopes using 40× (1.3NA, oil) or 60× (1.4NA, oil) objectives at 0.21 μm/px using NIS‐elements C software (Nikon).

### 4.9 DNA fiber labelling assay

Sorted HSPCs were incubated (60 min, 37°C) in RPMI‐1640 containing 20% FBS, 1% L‐glutamine, 1% penicillin/streptomycin, and 55 μM β‐mercaptoethanol, and then 5‐Iodo‐2′deoxyuridine (IdU, 25 μM) was added for 20 min at 37°C. Excess media was added, cells were centrifuged, then resuspended in 5‐Chloro‐2′‐deoxyuridine (CldU, 250 μM) containing media and incubated for 20 min at 37°C. Samples were blinded and DNA fibers were spread at room temperature, as previously described (Nieminuszczy et al., 2016; Puccetti et al., 2019). DNA was denatured for 60 min in 2.5 N HCl, washed in PBS, and blocked (2% BSA, 0.1% Tween‐20 in PBS) for 60 min. Slides were incubated with 1:200 rat anti‐BrdU/CldU (Abcam) and 1:50 mouse anti‐BrdU/IdU (BD) in blocking buffer for 2.5 hrs. After a PBS rinse, slides were incubated with goat anti‐rat AlexaFluor‐488 (1:250, Invitrogen) and goat anti‐mouse Cy3 (1:250, Invitrogen) in blocking buffer for 60 min. Slides were rinsed in PBS, air dried, mounted with ProLong Gold (Invitrogen), and cured overnight at room temperature. Images captured on a Nikon Eclipse N*i* with a 100× (1.45NA, oil) objective at 0.029 μm/px using a Nikon DS‐Qi2 camera and NIS Elements BR (Nikon) software. Analysis was performed with ImageJ (Fiji) software (Schindelin et al., 2012).

### 4.10 Comet assays

Neutral comet assays were performed on blinded samples as previously described (Puccetti et al., 2017; 2019) on isolated HSPC populations (see above). Images were captured on a Nikon Eclipse N*i* microscope with a 10× (0.45NA, air) objective at 0.879 μm/px using a Nikon DS‐Qi2 camera and NIS Elements BR (Nikon) software. Images were analyzed using CometScore 2.0 software.

### 4.11 Statistics

Data in Figures 1b,c, e–J and 3b–j; Figures S1A,B and S3H–I were assessed for normality by the Shapiro–Wilk test. Non‐normally distributed data were compared using the Mann–Whitney test (two‐tailed). If data were normally distributed, variance was assessed using the *F* test. Data with similar variance was evaluated using Student's *t*‐tests (two‐tailed). Data with unequal variance was compared using the *t*‐test with Welch's correction (two‐tailed). Also used were a two‐way ANOVA, chi‐squared test analysis for trend, and for fiber labeling binning graphs, Gaussian nonlinear regression curves were generated. Student's *t*‐tests were two‐tailed except for Figures 5g–i, 6e, k; Figure S5I–L,Q,V that were one‐tailed Student's *t*‐tests. All calculations were performed using GraphPad Prism software unless otherwise indicated. Statistical outliers as determined by Grubbs' (*α* = 0.05) or ROUT (*Q* = 1%) analysis were removed except for BMTs, γH2AX foci, and comet assay data. Statistical outliers in the 1:3 competitive BMT were determined by Grubbs' analysis (α = 0.001). Data in Figure 7a,k–m; Figure S7B,C,E–O were calculated by normalization of *Smarcal1* ^Δ/Δ^ mice to wild‐type mice (set at 1) and then DKO mice were normalized to *Smarcal1* ^Δ/Δ^ mice. All statistical tests used are indicated in the figure legend except those indicated above.

## AUTHOR CONTRIBUTIONS

Experiments were designed by SK, MVP, and CME. Experiments were performed by SK, MVP, CMA, IS, NJ, PM, and CME. Data analysis was performed by SK, MVP, IS, NJ, and BM. Figures were generated by SK and PM. Manuscript was written by SK and CME. All authors read, edited and approved the manuscript.

## CONFLICT OF INTEREST STATEMENT

Dr. Eischen had a sponsored research agreement with AbbVie that was independent of this project. The other authors declare no conflict of interest.

## Supporting information


Data S1.

Table S2.

## Data Availability

The cell‐based data from mouse bone marrow, spleen, and blood that support the findings of this study are available from the corresponding author upon reasonable request.
