# Supplementary material for: DNA fork remodeling proteins, Zranb3 and Smarcal1, are uniquely essential for aging hematopoiesis
Source: Aging Cell. 2024 Jul 23;23(11):e14281. doi: 10.1111/acel.14281 (PMC11561652; doi:10.1111/acel.14281)
Supplement: Supplementary file 1 — Data S1. [file ACEL-23-e14281-s001.pdf]

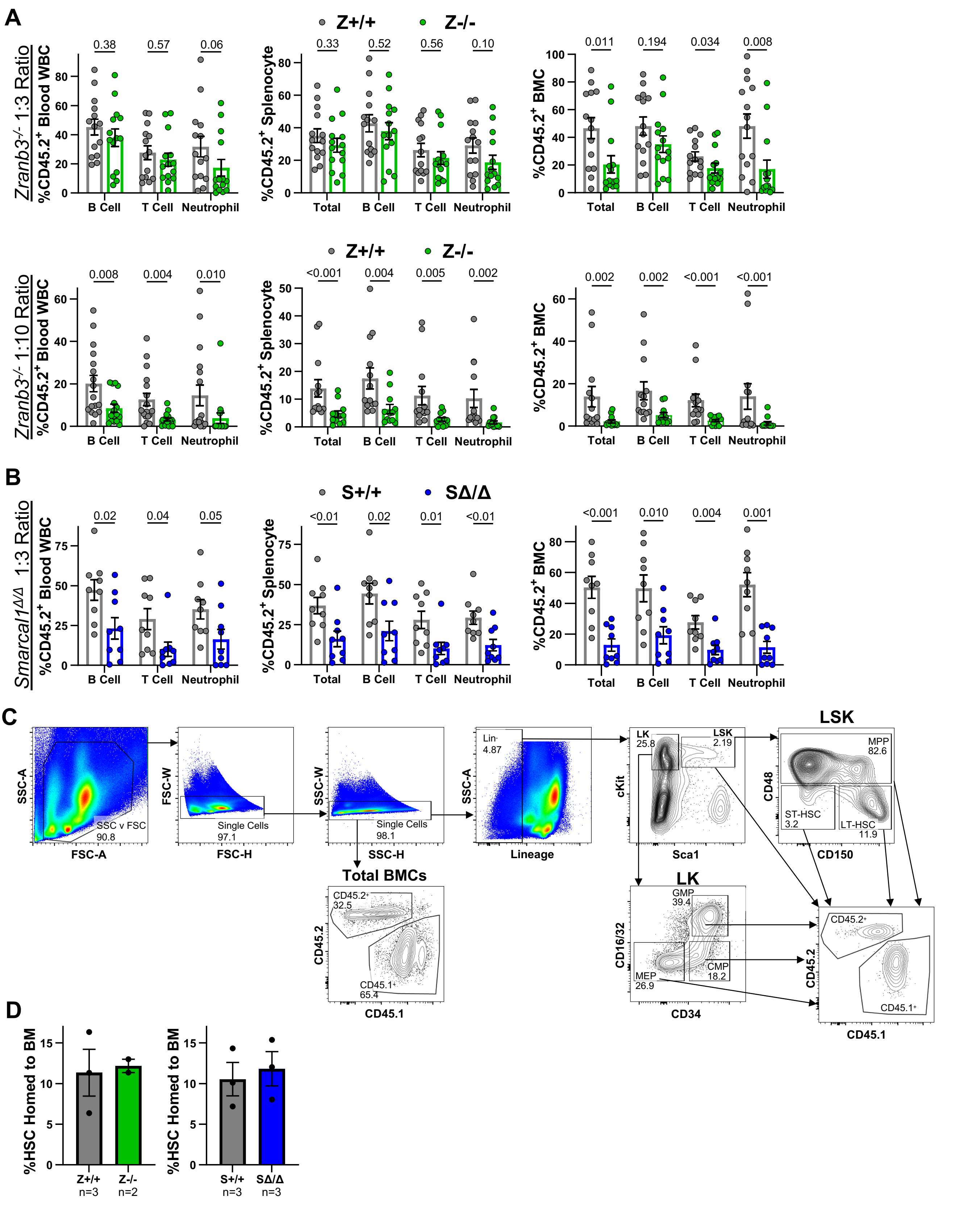
A
Z+/+
Z-/-
Zranb3-/- 1:3 Ratio
%CD45.2+ Blood WBC
%CD45.2+ Splenocyte
%CD45.2+ BMC
0.38
0.57
0.06
0.33
0.52
0.56
0.10
0.011
0.194
0.034
0.008
B Cell
T Cell
Neutrophil
Total
Zranb3-/- 1:10 Ratio
0.008
0.004
0.010
<0.001
0.004
0.005
0.002
0.002
0.002
<0.001
<0.001
B
S+/+
SΔ/Δ
Smarcal1Δ/Δ 1:3 Ratio
0.02
0.04
0.05
<0.01
0.02
0.01
<0.01
<0.001
0.010
0.004
0.001
C
SSC v FSC
90.8
Single Cells
97.1
Single Cells
98.1
Lin-
4.87
LK
25.8
LSK
2.19
MPP
82.6
ST-HSC
3.2
LT-HSC
11.9
Total BMCs
CD45.2+
32.5
CD45.1+
65.4
GMP
39.4
CMP
18.2
MEP
26.9
CD45.2+
CD45.1+
SSC-A
FSC-A
FSC-W
FSC-H
SSC-W
SSC-H
Lineage
cKit
Sca1
CD48
CD150
CD45.2
CD45.1
CD16/32
CD34
D
%HSC Homed to BM
Z+/+
n=3
Z-/-
n=2
S+/+
n=3
SΔ/Δ
n=3

Supplemental Figure S1. HSPC function is impaired upon Zranb3 and Smarcal1 loss, but they home to bone marrow. A, B) Hematopoietic cell populations of mice evaluated after competitive BMTs (see Figure 1A for schematic) with CD45.2^{+} BMCs from *Zranb3*$^{-/-}$ (A) or *Smarcal1*$^{\Delta/\Delta}$ (B) and their respective wild-type (WT) littermates mixed with WT CD45.1^{+} competitor BMCs at a ratio of 1(CD45.2):3(CD45.1) (A top, B) or 1(CD45.2):10(CD45.1) (A bottom). After 16 weeks, donor chimerism in blood (A and B left), spleen (A and B center), and bone marrow (A and B right) were measured. C) Representative HSPC gating strategy used for BMT recipients. D) Percentage of CFSE^{+} HSCs in bone marrow 24 hours after transplantation out of total CFSE^{+} HSCs transplanted. "n" denotes number of recipients; symbols denote individual recipients; mean ±SEM.

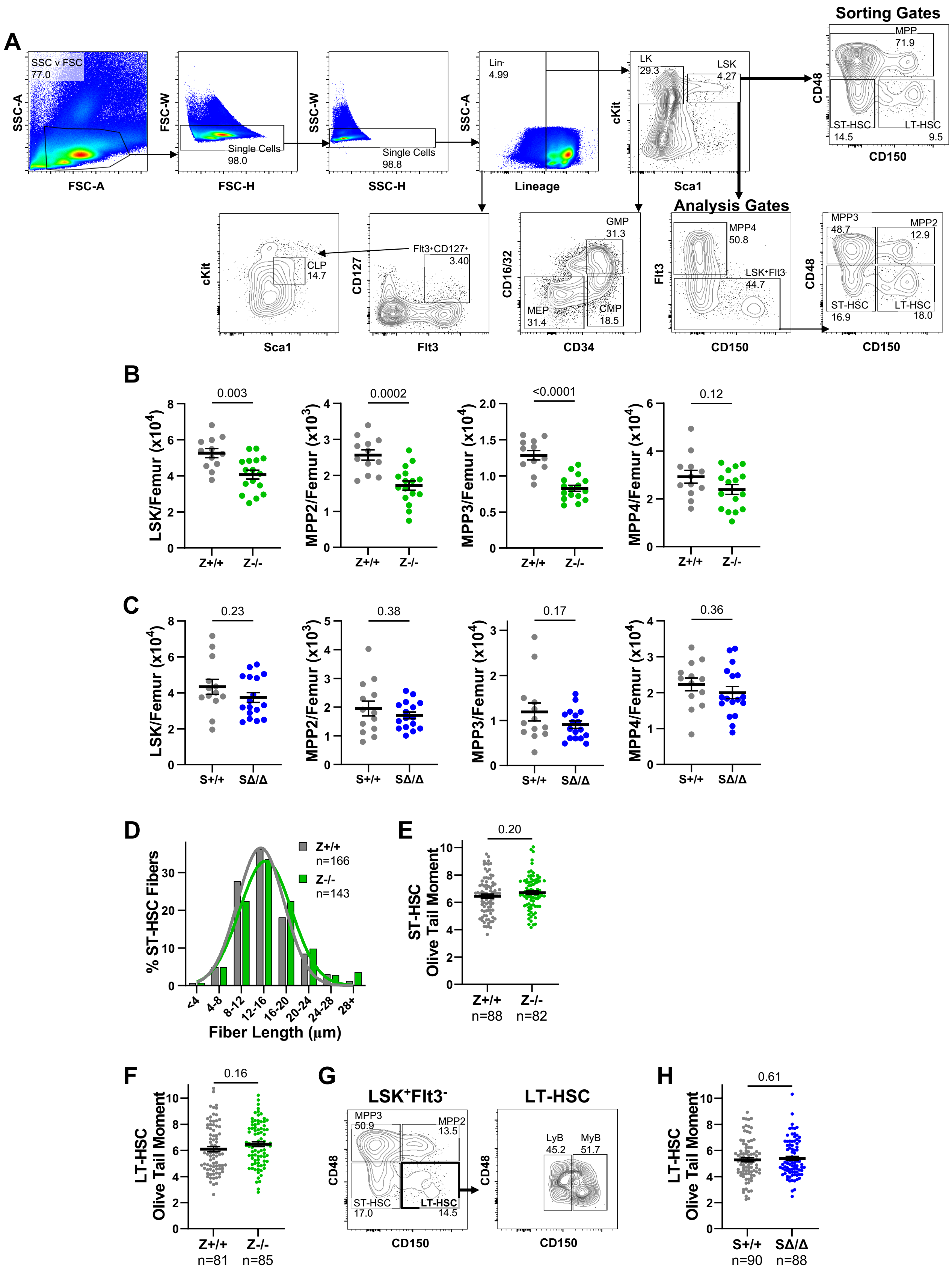
A
Sorting Gates
SSC v FSC
77.0
SSC-A
FSC-A
FSC-W
FSC-H
Single Cells
98.0
SSC-W
SSC-H
Single Cells
98.8
Lin-
4.99
Lineage
LK
29.3
LSK
4.27
cKit
Sca1
MPP
71.9
ST-HSC
14.5
LT-HSC
9.5
CD48
CD150
Analysis Gates
CLP
14.7
Flt3+CD127+
3.40
CD127
Flt3
GMP
31.3
MEP
31.4
CMP
18.5
CD16/32
CD34
MPP4
50.8
LSK+Flt3-
44.7
MPP3
48.7
MPP2
12.9
ST-HSC
16.9
LT-HSC
18.0
B
LSK/Femur (x10^4)
MPP2/Femur (x10^3)
MPP3/Femur (x10^4)
MPP4/Femur (x10^4)
0.003
0.0002
<0.0001
0.12
Z+/+
Z-/-
C
0.23
0.38
0.17
0.36
S+/+
SΔ/Δ
D
% ST-HSC Fibers
Z+/+
n=166
Z-/-
n=143
<4
4-8
8-12
12-16
16-20
20-24
24-28
28+
Fiber Length (μm)
E
ST-HSC
Olive Tail Moment
0.20
Z+/+
n=88
Z-/-
n=82
F
LT-HSC
Olive Tail Moment
0.16
Z+/+
n=81
Z-/-
n=85
G
LSK+Flt3-
MPP3
50.9
MPP2
13.5
ST-HSC
17.0
LT-HSC
14.5
LT-HSC
LyB
45.2
MyB
51.7
CD48
CD150
H
LT-HSC
Olive Tail Moment
0.61
S+/+
n=90
SΔ/Δ
n=88

Supplemental Figure S2. Defects in HSPCs and/or DNA replication stress arise without Zranb3 or Smarcal1. A) Representative gating strategy used to measure HSPC populations (Analysis Gates) or to sort LT-HSCs, ST-HSCs, and pooled MPPs (Sorting Gates) from non-transplanted mice (Pietras et al., 2015). B, C) Numbers of the indicated HSPC type/femur from young mice that are wild-type (+/+) or deficient in Zranb3 (B) or Smarcal1 (C); 12-17 mice/genotype; symbols denote individual mice; mean ±SEM; P-values determined by Student's t-test. D-H) ST-HSCs (D, E) or LT-HSC (F, H) isolated from mice of the indicated genotypes or measured (G) by flow cytometry from littermates (genotype indicated). Binning of DNA fiber lengths (D, "n" denotes number of fibers); curves are calculated Gaussian distribution. Comet assay Olive tail moments from ST-HSCs (E) and LT-HSCs (F, H). For E, F, and H, "n" denotes number of cells; symbols denote individual cells; mean ±SEM; P-values determined by Student's t-tests. Representative experiments shown for D-F and H. For G, Representative gating strategy used to measure "lymphoid-biased" (LyB) and "myeloid-biased" (MyB) LT-HSCs.

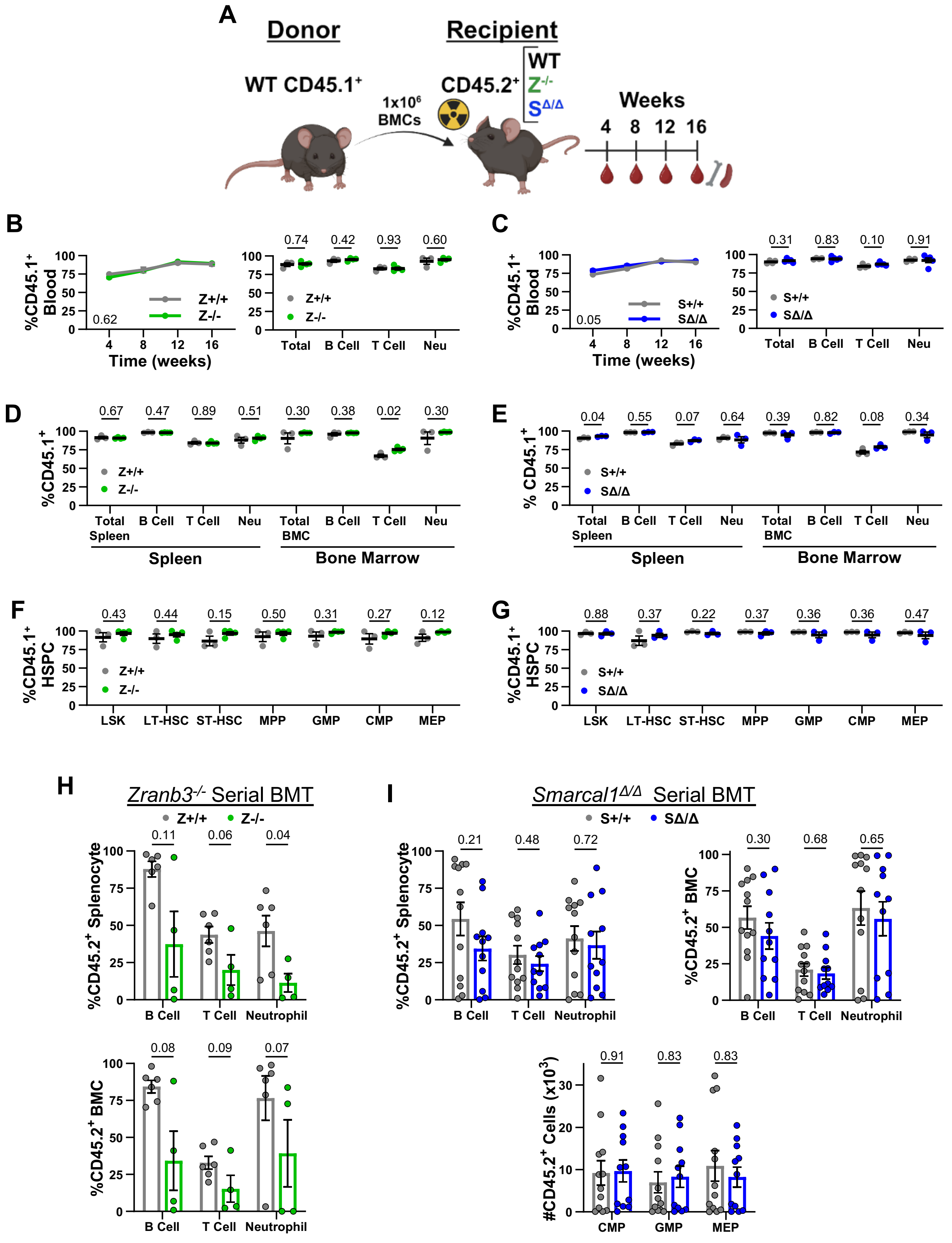

A
Donor
Recipient
WT CD45.1+
1x10^6 BMCs
CD45.2+
WT
Z-/-
SΔ/Δ
Weeks
4 8 12 16
B
%CD45.1+ Blood
Time (weeks)
Z+/+
Z-/-
0.62
0.74 0.42 0.93 0.60
Total B Cell T Cell Neu
C
S+/+
SΔ/Δ
0.05
0.31 0.83 0.10 0.91
D
%CD45.1+
0.67 0.47 0.89 0.51 0.30 0.38 0.02 0.30
Total Spleen B Cell T Cell Neu Total BMC B Cell T Cell Neu
Spleen
Bone Marrow
E
% CD45.1+
0.04 0.55 0.07 0.64 0.39 0.82 0.08 0.34
F
%CD45.1+ HSPC
0.43 0.44 0.15 0.50 0.31 0.27 0.12
LSK LT-HSC ST-HSC MPP GMP CMP MEP
G
0.88 0.37 0.22 0.37 0.36 0.36 0.47
H
Zranb3-/- Serial BMT
%CD45.2+ Splenocyte
0.11 0.06 0.04
B Cell T Cell Neutrophil
%CD45.2+ BMC
0.08 0.09 0.07
I
Smarcal1Δ/Δ Serial BMT
0.21 0.48 0.72
0.30 0.68 0.65
#CD45.2+ Cells (x10^3)
0.91 0.83 0.83
CMP GMP MEP

Supplemental Figure S3. Loss of Zranb3 or Smarcal1 cause HSPC intrinsic defects. A) Schematic of BMT to evaluate the bone marrow niche. B-G) CD45.1$^+$ total wild-type BMCs were transplanted into lethally-irradiated CD45.2$^+$ *Zranb3*$^{-/-}$ (B, D, F), *Smarcal1*$^{\Delta/\Delta}$ (C, E, G), or wild-type littermates of each. Donor chimerism in peripheral blood at 4-week intervals (B and C, left; mean ±SEM; P-value determined by two-way ANOVA). After 16 weeks post-transplant, donor chimerism in peripheral blood (B and C right), splenocytes (D and E, left), bone marrow (D and E, right) and HSPCs (F, G) were evaluated by flow cytometry (3-5 recipients/genotype; symbols denote individual mice; mean ±SEM; P-value determined by Student's t-tests). H, I) Hematopoietic cell populations of tertiary recipients post-serial BMT of BMCs from *Zranb3*$^{-/-}$ (H) or *Smarcal1*$^{\Delta/\Delta}$ (I) mice and their respective wild-type littermates. CD45.2$^+$ donor chimerism in spleen (H top, I top-left; 4-12 recipients/transplant), bone marrow (H bottom, I top-right; 4-12 recipients/transplant), and numbers of CD45.2$^+$ HSPCs/femur (I bottom; 7-12 recipients/transplant) were measured (symbols denote individual recipients; mean ±SEM; P-values determined as described in the methods).

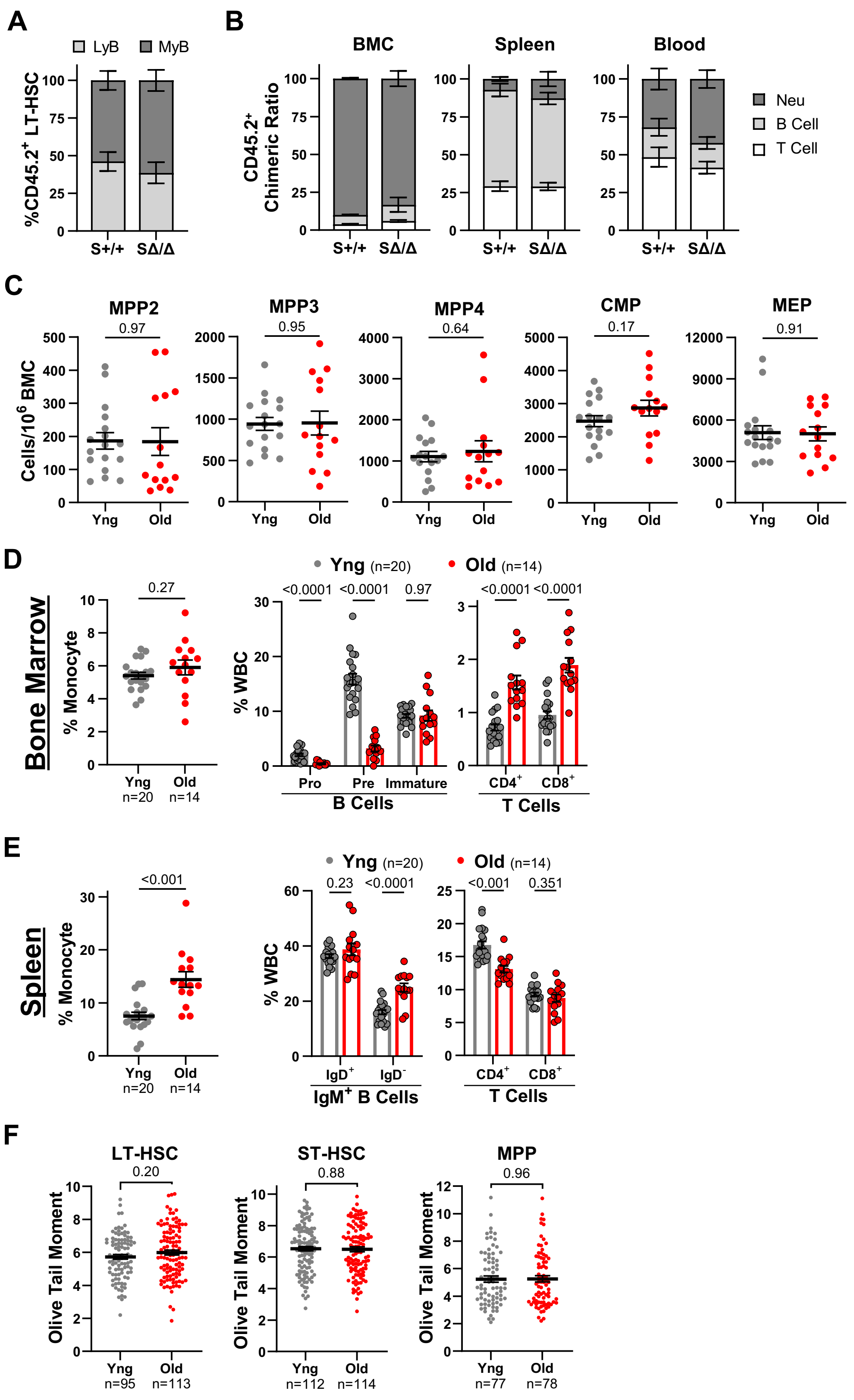
A
LyB
MyB
%CD45.2+ LT-HSC
S+/+
SΔ/Δ
B
BMC
Spleen
Blood
CD45.2+ Chimeric Ratio
Neu
B Cell
T Cell
C
MPP2
MPP3
MPP4
CMP
MEP
Cells/10^6 BMC
0.97
0.95
0.64
0.17
0.91
Yng
Old
D
Bone Marrow
% Monocyte
0.27
Yng (n=20)
Old (n=14)
% WBC
<0.0001
0.97
Pro
Pre
Immature
B Cells
CD4+
CD8+
T Cells
Yng n=20
Old n=14
E
Spleen
<0.001
0.23
0.351
IgD+
IgD-
IgM+ B Cells
F
LT-HSC
ST-HSC
MPP
Olive Tail Moment
0.20
0.88
0.96
Yng n=95
Old n=113
Yng n=112
Old n=114
Yng n=77
Old n=78

Supplemental Figure S4. LT-HSC functional exhaustion results in hematopoietic dysregulation A, B) Secondary transplant recipients of *Smarcal1*$^{\Delta/\Delta}$ and wild-type littermate control BMCs were evaluated by flow cytometry. The percentages of LyB-LT-HSCs and MyB-LT-HSCs out of total CD45.2^{+} LT-HSCs (A) and chimeric ratios of specific CD45.2^{+} populations in the bone marrow (B, left), spleen (B, center), and blood (B, right). For A, 6-10 recipients/genotype; for B (left and center), 3-7 recipients/genotype; for B (right) 8-12 recipients/genotype. C) The frequency of the indicated HSPC type per 10^{6} BMCs in young (Yng) and old wild-type mice (14-17 mice/age). D, E) Percentage of specific hematopoietic populations in the bone marrow (D) and spleen (E) from young or old wild-type mice; "n" denotes number of mice; symbols denote individual mice. F) Comet assay Olive tail moments from LT-HSCs (left), ST-HSCs (middle), and MPPs (right); "n" is number of cells; symbols denote individual cells; Representative experiments shown. For A-F, mean ±SEM; P-values determined by Student's t-tests.

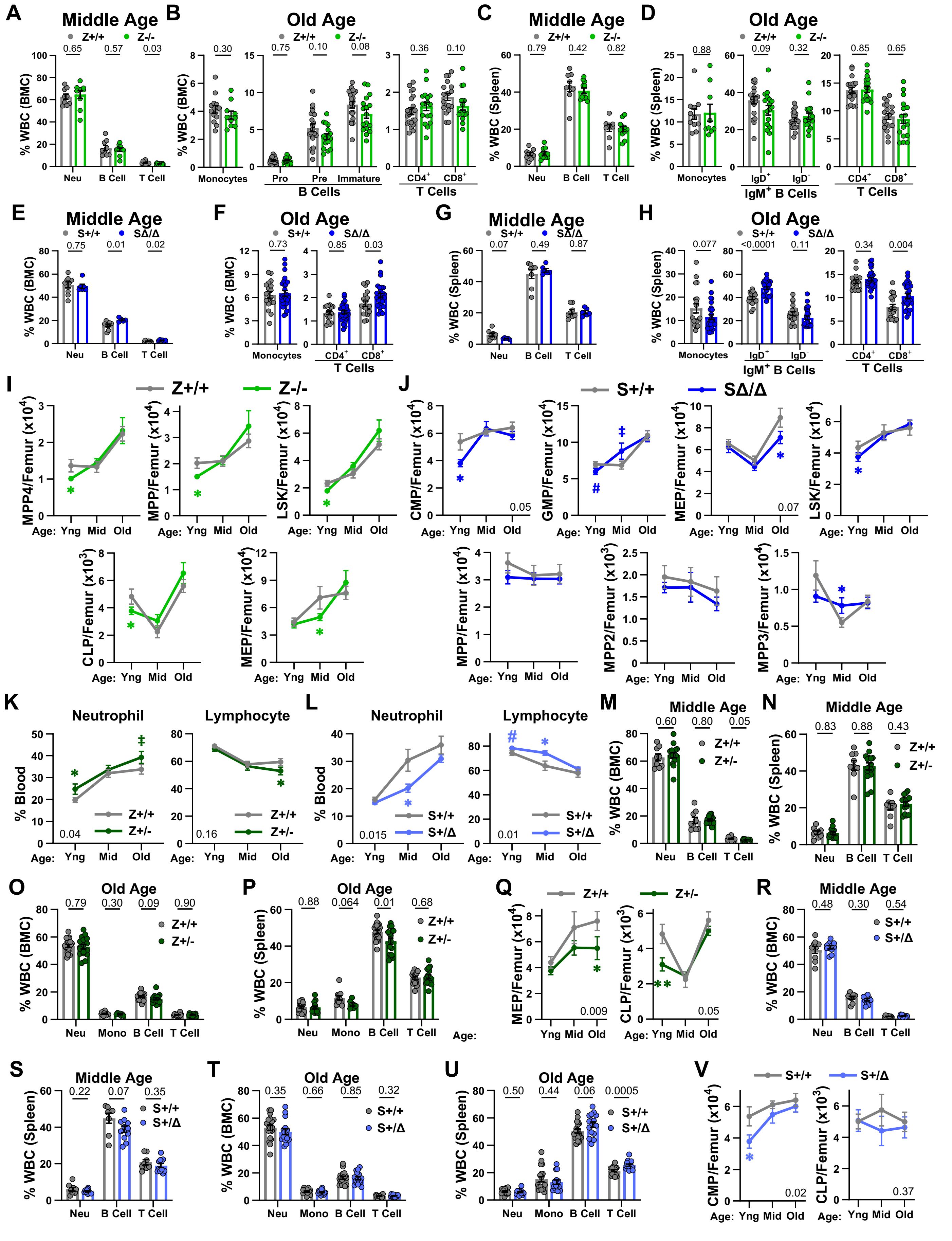
A
Middle Age
Z+/+ Z-/-
0.65 0.57 0.03
% WBC (BMC)
Neu B Cell T Cell
B
Old Age
Z+/+ Z-/-
0.30 0.75 0.10 0.08 0.36 0.10
% WBC (BMC)
Monocytes Pro Pre Immature CD4+ CD8+
B Cells
T Cells
C
Middle Age
Z+/+ Z-/-
0.79 0.42 0.82
% WBC (Spleen)
Neu B Cell T Cell
D
Old Age
Z+/+ Z-/-
0.88 0.09 0.32 0.85 0.65
% WBC (Spleen)
Monocytes IgD+ IgD- CD4+ CD8+
IgM+ B Cells
T Cells
E
Middle Age
S+/+ SΔ/Δ
0.75 0.01 0.02
% WBC (BMC)
Neu B Cell T Cell
F
Old Age
S+/+ SΔ/Δ
0.73 0.85 0.03
% WBC (BMC)
Monocytes CD4+ CD8+
T Cells
G
Middle Age
S+/+ SΔ/Δ
0.07 0.49 0.87
% WBC (Spleen)
Neu B Cell T Cell
H
Old Age
S+/+ SΔ/Δ
0.077 <0.0001 0.11 0.34 0.004
% WBC (Spleen)
Monocytes IgD+ IgD- CD4+ CD8+
IgM+ B Cells
T Cells
I
Z+/+ Z-/-
MPP4/Femur (x10^4)
MPP/Femur (x10^4)
LSK/Femur (x10^4)
CLP/Femur (x10^3)
MEP/Femur (x10^4)
Age: Yng Mid Old
J
S+/+ SΔ/Δ
CMP/Femur (x10^4)
0.05
GMP/Femur (x10^4)
MEP/Femur (x10^4)
0.07
LSK/Femur (x10^4)
MPP/Femur (x10^4)
MPP2/Femur (x10^3)
MPP3/Femur (x10^4)
Age: Yng Mid Old
K
Neutrophil
Lymphocyte
% Blood
Z+/+ Z+/-
0.04
0.16
Age: Yng Mid Old
L
Neutrophil
Lymphocyte
% Blood
S+/+ S+/Δ
0.015
0.01
Age: Yng Mid Old
M
Middle Age
0.60 0.80 0.05
Z+/+ Z+/-
% WBC (BMC)
Neu B Cell T Cell
N
Middle Age
0.83 0.88 0.43
Z+/+ Z+/-
% WBC (Spleen)
Neu B Cell T Cell
O
Old Age
0.79 0.30 0.09 0.90
Z+/+ Z+/-
% WBC (BMC)
Neu Mono B Cell T Cell
P
Old Age
0.88 0.064 0.01 0.68
Z+/+ Z+/-
% WBC (Spleen)
Neu Mono B Cell T Cell
Q
Z+/+ Z+/-
MEP/Femur (x10^4)
0.009
CLP/Femur (x10^3)
0.05
Age: Yng Mid Old
R
Middle Age
0.48 0.30 0.54
S+/+ S+/Δ
% WBC (BMC)
Neu B Cell T Cell
S
Middle Age
0.22 0.07 0.35
S+/+ S+/Δ
% WBC (Spleen)
Neu B Cell T Cell
T
Old Age
0.35 0.66 0.85 0.32
S+/+ S+/Δ
% WBC (BMC)
Neu Mono B Cell T Cell
U
Old Age
0.50 0.44 0.06 0.0005
S+/+ S+/Δ
% WBC (Spleen)
Neu Mono B Cell T Cell
V
S+/+ S+/Δ
CMP/Femur (x10^4)
0.02
CLP/Femur (x10^3)
0.37
Age: Yng Mid Old

Supplemental Figure S5. Aging mice lacking Zranb3 or Smarcal1 show hematopoietic dysregulation by unique phenotypes. A-D) Hematopoietic populations measured in the bone marrow (A, B) and spleen (C, D) of middle (A, C) and old (B, D) *Zranb3*$^{+/+}$ and *Zranb3*$^{-/-}$ mice. (≥9 mice/genotype.) E-H) Hematopoietic populations measured in the bone marrow (E-F) and spleen (G-H) of middle (E, G; 5-8 mice/genotype) and old (F, H; ≥19 mice/genotype) *Smarcal1*$^{+/+}$ and *Smarcal1*$^{\Delta/\Delta}$ mice. I, J) Numbers of the indicated HSPCs/femur of the indicated genotypes at the indicated age. (8-17 young, 6-10 middle age, and 8-14 old mice/genotype/population) K, L) Percentages of neutrophils (left) and lymphocytes (right) in the blood of mice of the indicated genotypes and age. (6-10 young, 8-20 middle age, and 11-16 old mice/genotype/population.) M-P) Hematopoietic populations measured in the bone marrow (M, O) and spleen (N, P) of middle age (M, N) and old (O, P) *Zranb3*$^{+/+}$ and *Zranb3*$^{+/-}$ mice. (≥6 mice/genotype). Q) Numbers of the indicated HSPCs/femur of the indicated genotypes and age (6-14 mice/genotype/age/population). R-U) Hematopoietic populations measured in the bone marrow (R, T) and spleen (S, U) of middle age (R, S) and old (T, U) *Smarcal1*$^{+/+}$ and *Smarcal1*$^{+/\Delta}$ mice. (≥8 mice/genotype.) V) Numbers of the indicated HSPCs/femur of the indicated genotypes and age. (5-13 mice/genotype/age/population.) For A-H, M-P, and R-U, mean ±SEM; symbols denote individual mice; P-values determined by Student's t-tests. For I-L, Q, and V, mean ±SEM; black P-values within graphs determined by 2-way ANOVA. P-values determined by Student's t-test at indicated ages are #P=0.07, ‡P=0.06, *P<0.05, **P<0.01.

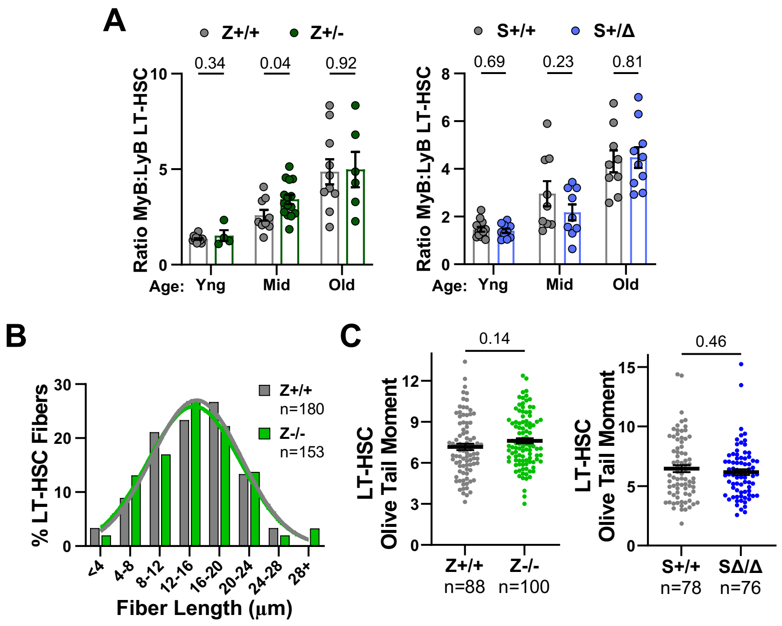
A
Z+/+
Z+/-
Ratio MyB:LyB LT-HSC
0.34
0.04
0.92
Age: Yng Mid Old
S+/+
S+/Δ
0.69
0.23
0.81
B
% LT-HSC Fibers
Z+/+
n=180
Z-/-
n=153
Fiber Length (μm)
C
LT-HSC
Olive Tail Moment
0.14
Z+/+
n=88
Z-/-
n=100
0.46
S+/+
n=78
SΔ/Δ
n=76

Supplemental Figure S6. Effects on LSK populations due to loss of Zranb3 or Smarcal1 are gene dose-dependent. A) Ratio of MyB-LT-HSCs to LyB-LT-HSCs of the indicated genotypes as measured by flow cytometry (4-13 young, 9-15 middle age, and 6-10 old mice/genotype/age; symbols denote individual mice; mean ±SEM; P-values determined by Student's t-test). B) Binning of DNA fiber lengths in isolated LT-HSCs from old mice (genotypes indicated, "n" denotes number of fibers; curves are calculated Gaussian distribution). C) Comet assay Olive tail moments from isolated LT-HSCs from old mice of indicated genotypes ("n" is number of cells; symbols denote individual cells; mean ±SEM; P-value determined by Student's t-tests). Representative experiments shown for B and C.

Kushinsky *et al*. Sup Fig S7

A

Genotype	# of Mice	% of Mice
Total	218	100.00%
$ZB^{+/+}$	60	27.52%
$ZB^{+/-}$	102	46.79%
$ZB^{-/-}$	56	25.69%

Genotype	# of Mice	% of Mice
Total	204	100.00%
$SM^{+/+}$	49	24.02%
$SM^{+/\Delta}$	98	48.04%
$SM^{\Delta/\Delta}$	57	27.94%

Genotype	# of Mice	% of Mice
Total	222	100.00%
$ZB^{+/+}SM^{\Delta/\Delta}$	49	22.07%
$ZB^{+/-}SM^{\Delta/\Delta}$	117	52.70%
$ZB^{-/-}SM^{\Delta/\Delta}$	56	25.23%

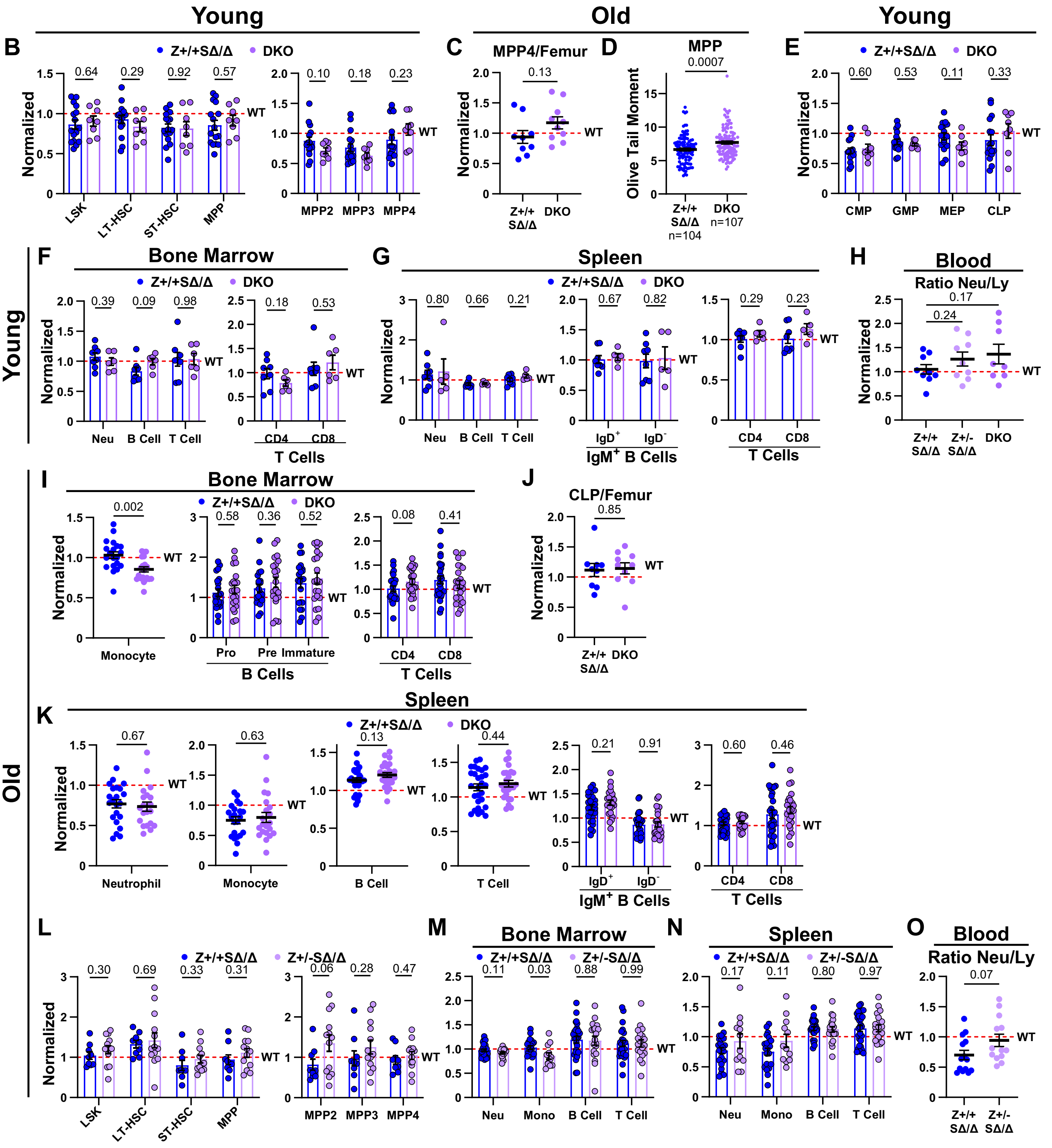

Supplemental Figure S7. Simultaneous loss of Zranb3 and Smarcal1 does not affect the survival of the offspring born but does impact hematopoiesis. A) Genotypes of offspring born from breeding heterozygous to heterozygous (*Zranb3*$^{+/-}$, *Smarcal1*$^{+/-}$, or *Zranb3*$^{+/-}$*Smarcal1*$^{\Delta/\Delta}$). B, C) Normalized numbers of specific HSPCs/femur from young (B) and old (C) *Zranb3*$^{+/+}$*Smarcal1*$^{\Delta/\Delta}$ and *Zranb3*$^{-/-}$*Smarcal1*$^{\Delta/\Delta}$ (DKO) mice. D) Comet assay Olive tail moments from isolated MPPs from old mice ("n" is number of cells; symbols denote individual cells; mean ±SEM; P-value determined by Student's t-tests). E-K) Hematopoietic populations measured in young (E-H) or old (I-K) mice of the indicated genotypes. Normalized numbers of HSPC/femur (E, J). Normalized values of percentages of hematopoietic cells from the bone marrow (F, I) or spleen (G, K). Normalized values of the ratio of neutrophils to lymphocytes in the blood (H). L-O) Hematopoietic populations measured in old *Zranb3*$^{+/+}$*Smarcal1*$^{\Delta/\Delta}$ and *Zranb3*$^{+/-}$*Smarcal1*$^{\Delta/\Delta}$ mice. Normalized numbers of HSPC/femur (L). Normalized values of percentages of hematopoietic cells from the bone marrow (M) or spleen (N). Normalized values of the ratio of neutrophils to lymphocytes in the blood (O). For B-O, except D, data normalized to values of wild-type mice (dashed line) which were set at one. Symbols denote individual mice; mean ±SEM; P-values determined by Student's t-tests. For young mice (B, E-H), 5-16 mice/genotype/population. For old mice (C, I-O), ≥9 mice/genotype.

Table S1. Summary of results from young mice lacking Zranb3 or Smarcal1 compared to littermate wild-type controls.

Population	Genotype	DNA Damage (γH2AX Foci)	DNA Replication Stress	DNA Fiber Length	DNA Breaks (Comet Assay)	Cell Numbers	Ratio LyB:MyB LT-HSCs
MPP	Zranb3	Increase	Increase	Increase (Fork Bypass)	Increase	Decrease	N/A
	Smarcal1	Increase	Increase	Decrease (Fork Stalling)	No Change	Slight Trend Decrease	N/A
ST-HSC	Zranb3	Increased	No Change	No Change	No Change	Trend Decrease	N/A
	Smarcal1	Increased	Increase	Decrease (Fork Stalling)	Increase	Trend Decrease	N/A
LT-HSC	Zranb3	Increased	Increase	Decrease (Fork Stalling)	No Change	Trend Decrease	Reduced LyB Increased MyB
	Smarcal1	No Change	Not Determined	Not Determined	No Change	Normal	Normal
